# Supplementary material for: Histone H4 lysine 16 acetylation controls central carbon metabolism and diet-induced obesity in mice
Source: Nat Commun. 2021 Oct 27;12:6212. doi: 10.1038/s41467-021-26277-w (PMC8551339; doi:10.1038/s41467-021-26277-w)
Supplement: Supplementary file 1 — Supplementary Info [file 41467_2021_26277_MOESM1_ESM.pdf]

Supplementary Information

Histone H4 lysine 16 acetylation controls central carbon metabolism and diet-induced obesity in mice

Cecilia Pessoa Rodrigues[1,2,3*], Aindrila Chatterjee[1,4*], Meike Wiese[1], Thomas Stehle[1], Witold Szymanski[5], Maria Shvedunova[1], Asifa Akhtar[1,2,#]

1. Department of Chromatin Regulation, Max Planck Institute of Immunobiology and Epigenetics, 79108 Freiburg, Germany.
2. University of Freiburg, Faculty of Biology, Schaenzlestrasse 1, 79104 Freiburg, Germany.
3. International Max Planck Research School for Molecular and Cellular Biology (IMPRS-MCB), Freiburg, Germany.
4. European Molecular Biology Laboratory, Meyerhofstrasse 1, 69117 Heidelberg, Germany
5. Proteomics Facility, Max Planck Institute of Immunobiology and Epigenetics, Freiburg, Germany

* share equal contribution

# Corresponding author
akhtar@ie-freiburg.mpg.de
Phone: +49 (0)7615108565
Fax: +49 (0)761510856

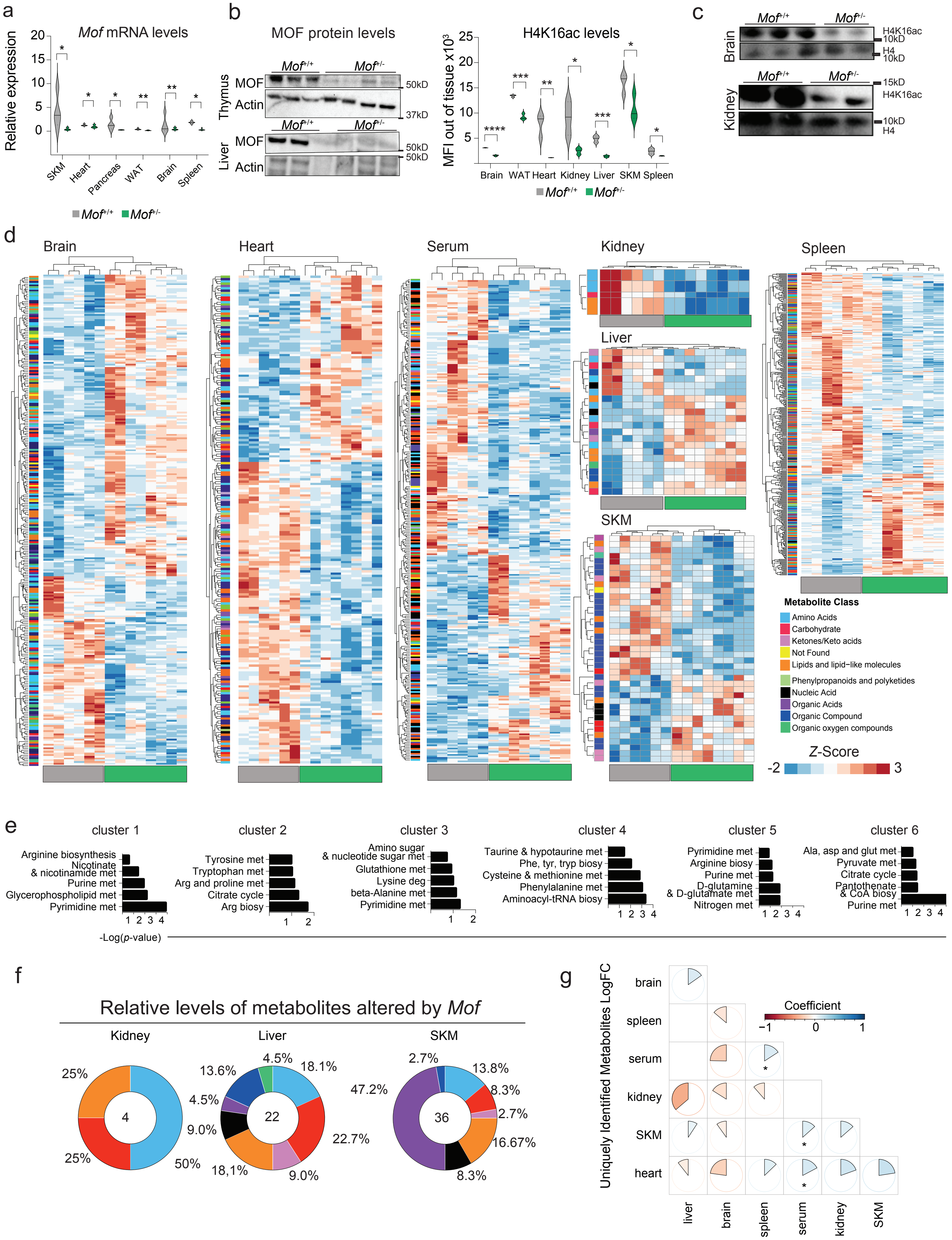

Supplementary Figure 1

Supplementary Figure Legends

Supplementary Fig. 1. Metabolic characterisation of seven distinct organs of *Mof*$^{+/-}$ mice. Related to Fig. 1

(a) RT-qPCR analyses in SKM, pancreas and visceral WAT of *Mof*$^{+/+}$ (grey) and *Mof*$^{+/--}$ mice (green). Average *Mof* mRNA expression of biological replicates (*n*=4) is shown relative to *Hprt*. Statistical analysis was performed using a two-sided Mann-Whitney test. *p=0.05, **p=0.007. Dotted lines show the quartiles and solid lines depict the medians.
(b) Left: Immunoblotting of MOF in thymus and liver tissue of *Mof*$^{+/-}$ and *Mof*$^{+/+}$ mice. β-Actin was used as a loading control. Each lane represents a biological replicate. Right: Violin-plots showing the H4K16ac levels determined by FACS staining of different tissues. MFI: Median fluorescence intensity. Number of animals *n*=4. Statistical analysis was performed using two-sided t-test test, *p=0.02; **p=0.001;***p=0.0003; ****p=0.000019. Dotted lines show the quartiles and solid lines depict the medians.
(c) Immunoblotting of H4K16ac in brains and kidneys of *Mof*$^{+/-}$ and *Mof*$^{+/+}$ mice. H4 was used as a loading control. Each lane represents a biological replicate.
(d) Heatmaps showing the *Z*-Scores for significantly deregulated metabolites (DMs) found in the indicated organs. Metabolic classes of individual metabolites are indicated on the left and colour coded. DMs showing similar trends were aggregated and the hierarchical dendrograms are shown on the left side of the heatmaps.
(e) KEGG enrichment test for the unsupervised *k*-means clusters. Significantly enriched pathways are highlighted in red. Significant enrichment was scored by a two-sided Fisher test using *p*-value <0.05. *See also: Fig.1c*.
(f) Pie-charts showing the percentage of metabolic classes of DMs (q<0.1) in indicated *Mof*$^{+/-}$ tissues. Metabolite classes are color coded as depicted in (c). Total number of DMs per tissue are shown in the center of the pie-chart.
(g) Pie-plots showing the correlation coefficient of uniquely identified metabolites per tissue. Number of metabolites is indicated by the size of the pie slice. Positive correlation (blue) and anti-correlation (red) are indicated. The statistical significance was tested by the two-sided Pearson correlation with a *P*-value cutoff of p=0.05.

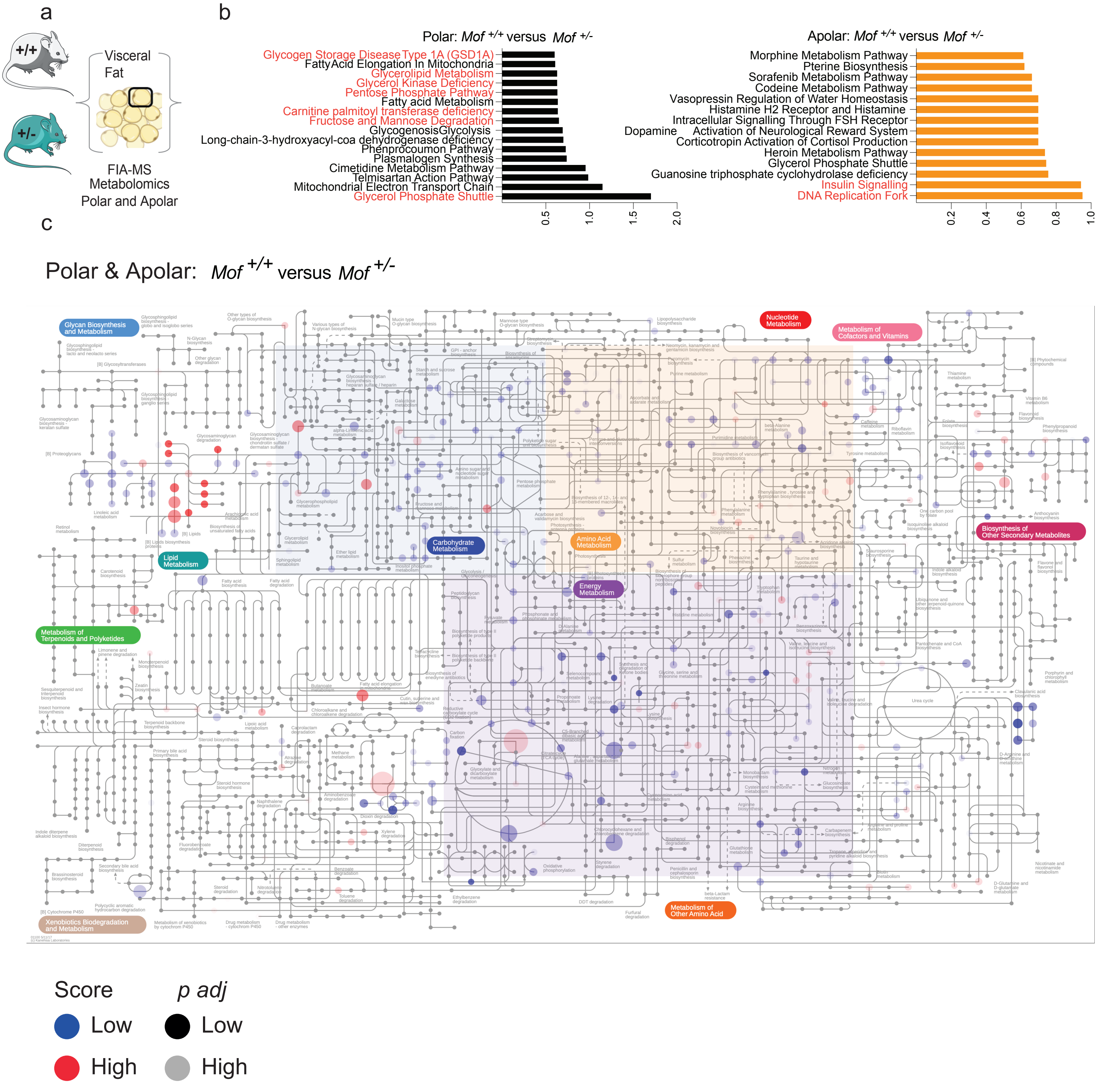

Supplementary Figure 2

Supplementary Fig. 2. Visceral adipocyte polar and apolar metabolite profile from *Mof*$^{+/-}$ and *Mof*$^{+/+}$ mice.

(a) Graphic representation of the preparation of visceral adipocytes for metabolomic analyses. *Mof*$^{+/+}$ *n*=3 and *Mof*$^{+/-}$ *n*=4.
(b) Barplots showing the enriched KEGG pathways in *Mof*$^{+/-}$ mice in polar (black) and apolar (orange) metabolites. False discovery rates (FDR) were controlled by the Benjamini-Hochberg (BH) method having q-value defined as 0.05.
(c) Metabolic pathway overview from metabolites scored in the visceral adipocyte metabolics. Color indicates the score value and intensity depicts *p-adjust* value. False discovery rates (FDR) were controlled by the Benjamini-Hochberg (BH) method having a *q*-value defined as 0.05.

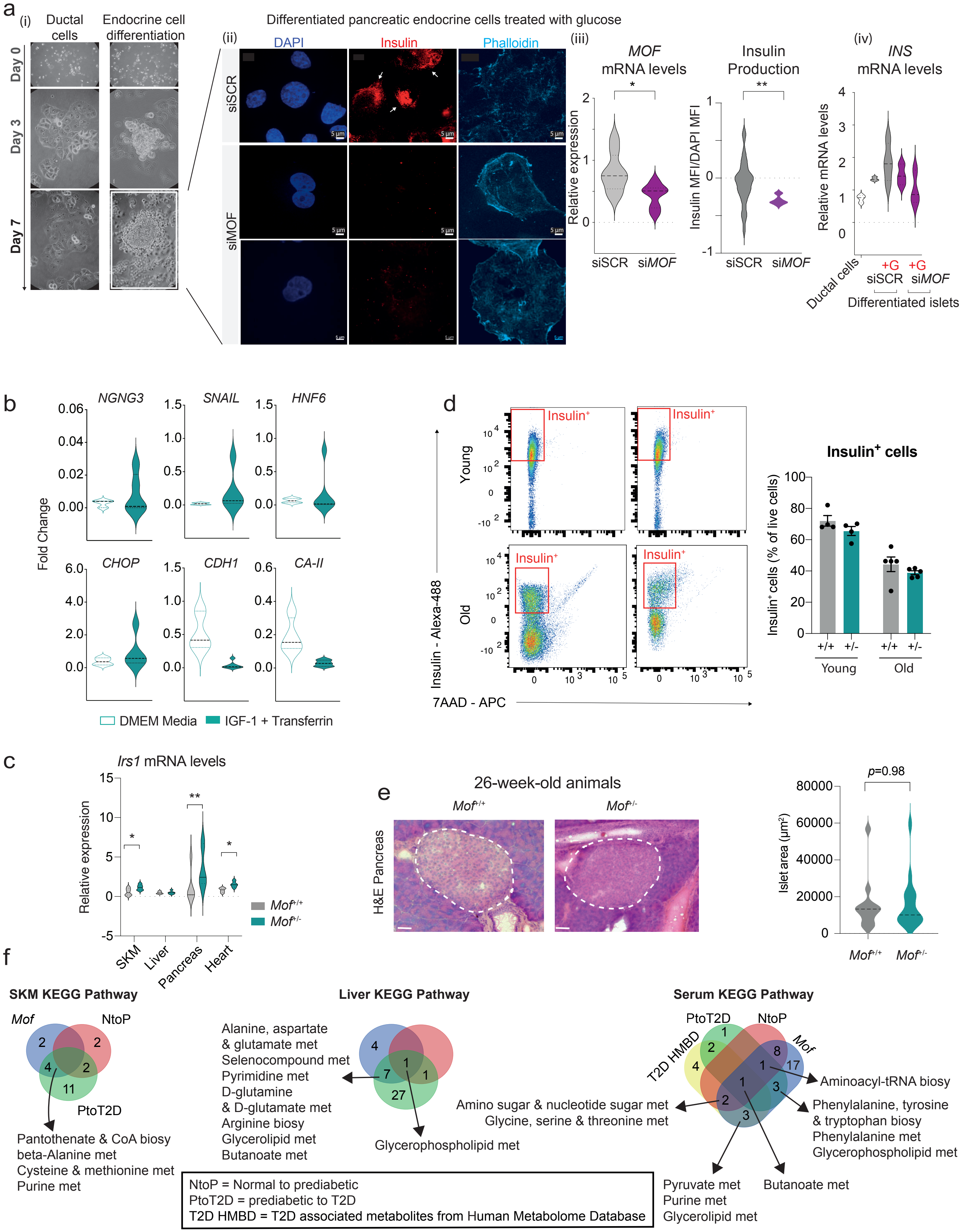

Supplementary Figure 3

Supplementary Fig. 3. Associating the metabolic profile of *Mof*$^{+/-}$ animals with the onset of metabolic disorders. Related to Fig. 3

(a) Representative images of (i) in vitro differentiated human pancreatic endocrine cells (Panc1) treated with (ii) control and *MOF*-targeting siRNAs and stained for insulin (red) post glucose stimulation. Co-staining with phalloidin (purple) and DAPI (blue). Scale bar: 5 µm. (iii) RT-qPCR analyses of *Mof* levels and Log-Relative median fluorescence intensity quantification for insulin ratio signal (Insulin MFI/DAPI MFI). (iv) Ductal cells before (white) and after differentiation in control (gray) or *MOF* siRNA-treated (purple) cells. Glucose stimulation is indicated in red "+G". Average insulin mRNA expression relative to *HPRT; n*=3 biological replicates. Statistical analysis was performed by t-test, *p=0.05, **p=0.023. For panels i, ii and iv, technical replicates (n=5) from 2 independent experiments with similar results are shown.

(b) RT-qPCR analysis in vitro differentiated islets upon IGF-1 and transferrin treatment used to induce differentiation (teal). Violin-plots show mRNA expression for epithelial to mesenchymaltransition-related genes in differentiated islets relative to *C1orf43* (n=3 biological replicates). Dotted lines inside the violin plots show the quartiles and dashed lines depict the medians.

(c) RT-qPCR analyses for *Irs1* levels in SKM, pancreas, heart and liver of *Mof*$^{+/+}$ (grey) and *Mof*$^{+/-}$ mice (green). Average *Isr1* mRNA expression of biological replicates (n=4) is shown relative to *Hprt*. Statistical analysis was performed using two-sided Mann-Whitney test, *p=0.0286, **p=0.0026. Dotted lines inside the violin plots show the quartiles and solid lines depict the medians.

(d) Dot-plot displaying the frequency of insulin-producing cells in the pancreas. Quantification is shown on the bar-plot (n=4). Error bars represent means ±SEM.

(e) Left: Pancreatic morphological evaluation based on hematoxylin-eosin (H&E) staining. Scale bar: 500 µm. Right: Quantification of islet area in aged mice. Images were acquired using the 40x objective and the number of islets was manually counted and area calculated for each individual islet. Number of islets *Mof*$^{+/+}$ n=12 and *Mof*$^{+/-}$ n=16 from n=4 mice per genotype. Statistical analysis was performed by Mann-Whitney test. p-value is indicated in the Figure. Dashed lines inside the violin plots show the medians.

(f) Left: The overlap of KEGG pathways enriched for the DM found in SKM of *Mof*$^{+/-}$ mice, differentially regulated upon normal to pre-diabetic states (NtoP) and pre-diabetic to type 2 diabetic patients. Middle: The overlap of KEGG pathways enriched for the DM found in liver of *Mof*$^{+/-}$ mice, differentially regulated upon normal to NtoP and PtoN. Right: The overlap of KEGG pathways enriched for the DM found in serum of *Mof*$^{+/-}$ mice, differentially regulated upon normal to NtoP, PtoN and T2D-associated metabolites from the Human Metabolome Database (HMBD). The arrows indicate pathways which were frequently found within the datasets.

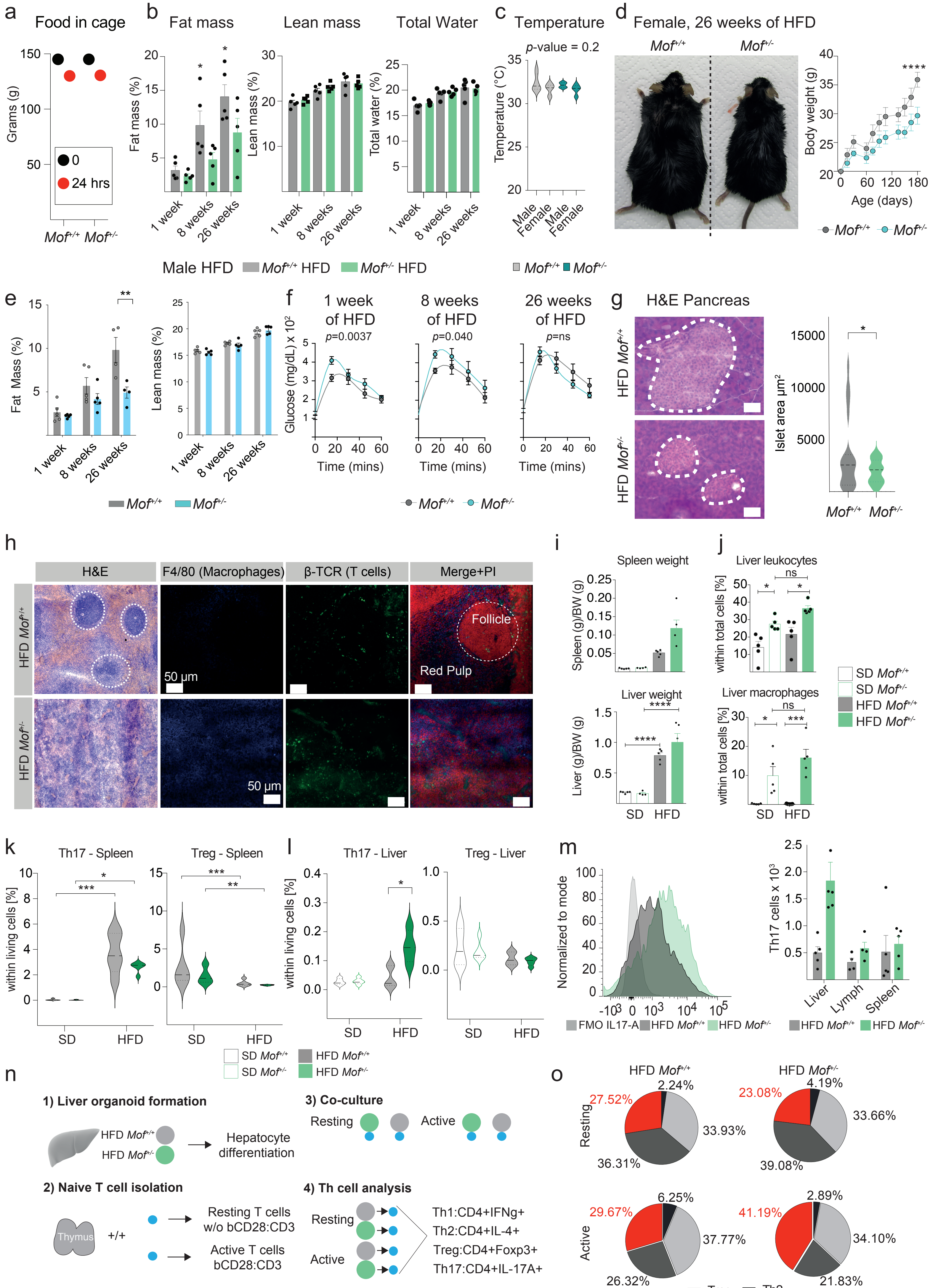

Supplementary Figure 4

Supplementary Fig. 4. Global metabolic characterisation of wildtype and *Mof*$^{+/-}$ animals on a high fat diet (HFD). Related to Fig. 3

(a) The grams of high-fat diet food present in a cage was weighed at time point 0 and after 24 hours. Number of animals *n*=4; mice *n*=7. *P*-value determined by Mann-Whitney test.
(b) Characterisation of animal body composition on HFD. Fat mass, lean mass and total water composition of male animals on HFD. Dots in the bar plot represent independent animals. *n*=5. t, *P*-value determined by two-sided two-way ANOVA followed by the Sidak's multiple comparison test, left *p=0.03 and right *p=0.016. Error bars show ±SEM.
(c) Abdominal temperature of *Mof*$^{+/+}$ (grey) and *Mof*$^{+/-}$ (teal) animals. Male (*Mof*$^{+/+}$ *n*=7 and *Mof*$^{+/-}$ *n*=4), Female (*Mof*$^{+/+}$ *n*=7 and *Mof*$^{+/-}$ *n*=4). Three measurements from the same animal were taken with an interval of ±15 seconds. *P*-value was determined by one-way ANOVA test. Dotted lines inside the violin plots show the quartiles and dashed lines depict the medians.
(d) Left: Representative images of control and *Mof* heterozygous female mice after 26 weeks of HFD. Right: Body weight gain under HFD. Statistical analysis performed by two-sided two-way ANOVA, ****$p=10^{-16}$, number of animals *n*=5. Error bars show ±SEM.
(e) Total fat mass (left) and lean mass (right) of 26-week-old female animals on HFD. Statistical analysis performed by two-way ANOVA, followed by Holm-Sidak's comparison test, **p=0.005, *n*=5. Error bars show ±SEM.
(f) Glucose tolerance test screening of female mice at 1, 8 and 26 weeks on HFD. Diet change was initiated at 7 weeks of age. Statistical analysis performed by two-sided two-way ANOVA, followed by Holm-Sidak's comparison test. *P*-values are shown in the Figure, *n*=5. Error bars show ±SEM.
(g) Left: Morphological evaluation of pancreas based on hematoxylin-eosin (H&E) staining of HFD animals (Representative image out of 4 independent biological replicates). Right: Islet area quantification of 27-week-old mice fed a HFD. Biological replicates *n*=4. Images were acquired using the 40x objective and the number of islets evaluated, and the number of islets was manually counted and area calculated for each individual islet. Number of quantified islets *Mof*$^{+/+}$ = 23 and *Mof*$^{+/-}$ = 25. Scale bar: 500 µm. Statistical analysis performed by two-sided Mann-Whitney test, *p= 0.021. Dotted lines inside the violin plots show the quartiles and dashed lines depict the medians
(h) Morphological evaluation of spleen based on hematoxylin-eosin (H&E) staining (left), immunofluorescence showing the global splenic immune architecture. Red pulp defined by tissue resident macrophages (F4/80+ cells, depicted in blue), periarteriolar lymphoid sheath (PALMS), characterised as the T cells (β-TCR+, depicted in green) region within the white pulp and nucleus staining shown in red. The circles indicate a follicle. Scale bar, 50 µm. Representative image from 3 independent animals showing similar results.
(i) Upper: Spleen weight ratio of 26-week-old animals fed SD (*Mof*$^{+/+}$ *n*=5; *Mof*$^{+/-}$ *n*=4) or HFD. Dots in the graph indicate individual animals. Lower: Liver weight ratio (Liver weight (g)/total body weight (g)) of 26-week-old animals under SD (*Mof*$^{+/+}$ *n*=5; *Mof*$^{+/-}$ *n*=4) or HFD (*Mof*$^{+/+}$ *n*=5; *Mof*$^{+/-}$ *n*=5). Dots in the graph indicate individualf animals. Statistical analysis performed by two-way ANOVA, followed by Tukey comparison test. ****$p=10^{-16}$. Error bars show ±SEM.
(j) Bar-plots showing the frequencies within the living cells of hepatic leukocytes (upper) and hepatic macrophages (lower). The diet condition is shown on the x-axis. Genotype follows the color code as: *Mof*$^{+/+}$, grey bars and *Mof*$^{+/-}$, green bars. Each dot represents an individual animal, *n*=5. Statistical analysis performed by two-way ANOVA, followed by Holm-Sidak's comparison test, comparison between *Mof*$^{+/+}$ *and Mof*$^{+/-}$ at of SD *p=0.03; and at 26 weeks of HFD *p=0.016, ***p=0.0001. Error bars show ±SEM.

(k) Frequency of viable Th17+ (left) or T regulatory cells (Treg) cells (right) found in spleen upon SD (*Mof*$^{+/+}$ *n*=5; *Mof*$^{+/-}$ *n*=4) or HFD (*Mof*$^{+/+}$ *n*=5; *Mof*$^{+/-}$ *n*=4). Statistical analysis performed by two-way ANOVA, followed by Holm-Sidak's comparison test, **p*=0.013, ***p*=0.0011; ****p*=0.00005. Dotted lines inside the violin plots show the quartiles and dashed lines depict the medians.

(l) As **(k)** but showing the hepatic frequencies. Statistical analysis performed by two-way ANOVA, followed by Holm-Sidak's comparison test, **p*=0.0301

(m) Left: Representative histogram showing the IL17 medium intensity fluorescent (MFI) of hepatic CD4+ T cells after 26 weeks on HFD (*Mof*$^{+/+}$, black; *Mof*$^{+/-}$, green). Fluorescence minus one (FMO) control is shown in grey. Right: Barplots showing the quantification of CD4+IL17+ (Th17) cells in the liver, inguinal lymph nodes and spleen. *n*=5 for liver/spleen and *n*=4 for inguinal lymph nodes. Each dot represents an individual animal. Error bars show ±SEM.

(n) Schematic representation of the organoid formation and in vitro co-culture. Numbers represent the order of events.

(o) Pie-charts showing the immune phenotype characterisation after co-culture. 3 organoids generated from 3 independent animals of each genotype were used for the co-culture.

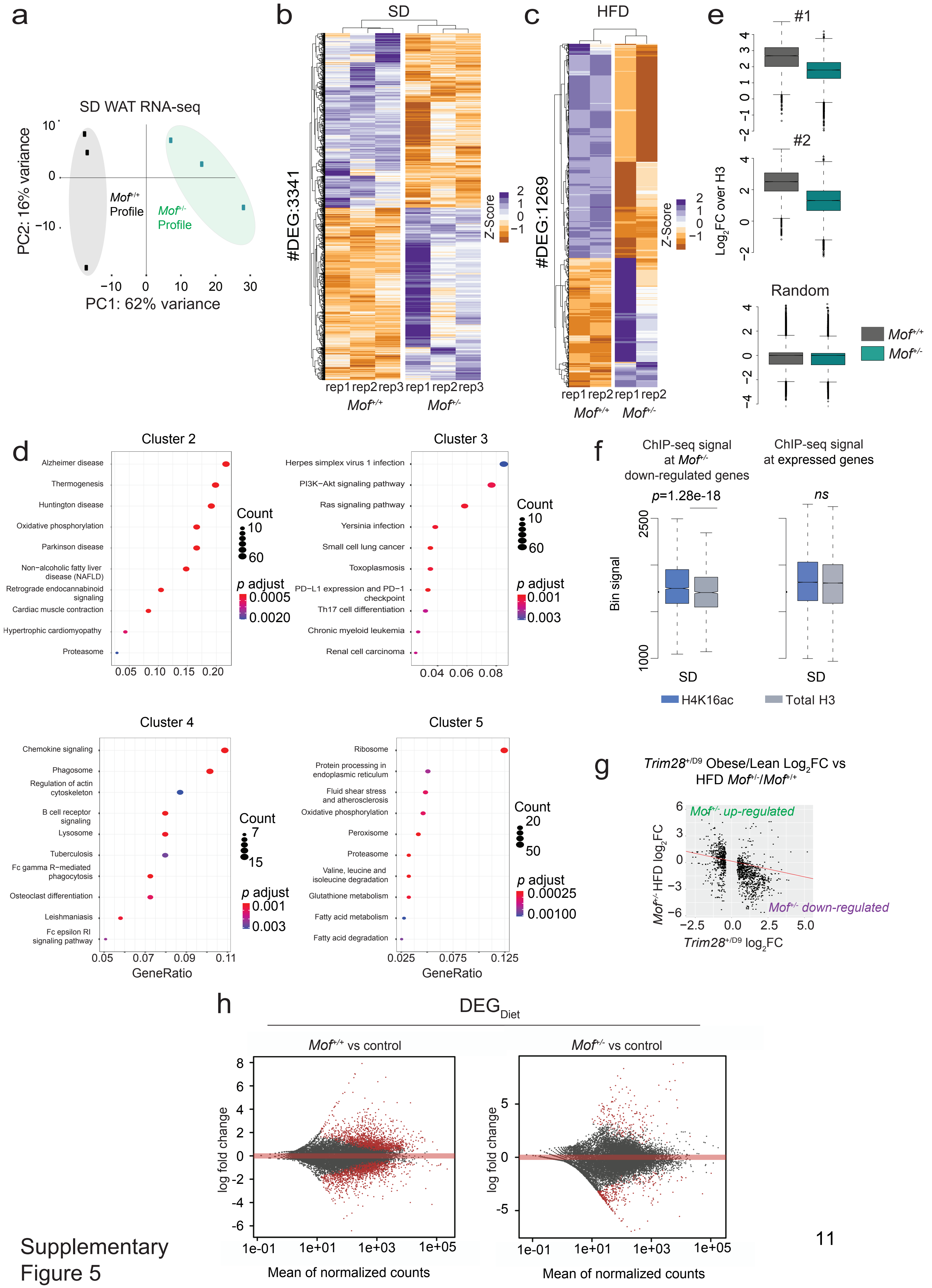

Supplementary Figure 5

Supplementary Fig. 5. Transcriptional characterisation of *Mof*$^{+/-}$ WAT. Related to Fig. 4.

(a) PCA-plot showing the transcriptional similarities between white adipocyte tissue (WAT) from wildtype (*Mof*$^{+/+}$, black dots) and *Mof* heterozygous (*Mof*$^{+/-}$, green dots) mice.
(b) Heatmap showing the transcript expression pattern of differentially expressed genes (DEG) of 8-week-old animals. Scale shows transcript *Z*-score.
(c) Heatmap showing the transcript expression pattern of differentially expressed genes (DEG) after 26 weeks on HFD. Scale shows transcript *Z*-score.
(d) KEGG enriched pathway for cluster 2, cluster 3 (found specifically upregulated in the HFD *Mof*$^{+/+}$ animals), cluster 4 and cluster 5. Circle size represents the number of DEG associated with the pathway. *P*-value is represented by the color scale indicated in the Figure. False discovery rates (FDR) were controlled by the Benjamini-Hochberg (BH) method with q-value defined as 0.05.
(e) Boxplots displaying the MOF ChIP enrichment intensity of MACS2 peaks per cluster in *Mof*$^{+/+}$ (grey) and *Mof*$^{+/-}$ (green) datasets. Enrichment scores were calculated using deepTools multibigwigsummary. 20,000 random genomic regions were used to validate MOF ChIP specificity. The two 'hinges 'are versions of the first and third quartile, i.e., close to quantile(x, c(1,3)/4). The hinges equal the quartiles for odd *n* (where n <- length(x)) and differ for even *n*. The plot was generated using the boxplot() function in R.
(f) Boxplot displaying the H4K16ac (blue) and H3 (grey) ChIP enrichment intensities of downregulated genes in *Mof*$^{+/-}$ WAT (mm10 genome). Enrichment scores were calculated using deepTools2 multiBigwigSummary. Statistical analysis was performed by a two-sided Welch test (ns: not significant). Original ChIP-seq data from [62]. The hinges equal the quartiles for odd *n* (where n <- length(x)) and differ for even *n*. The plot was generated using the boxplot() function in R.
(g) Scatter-plot showing the correlation between the WAT Log_2 fold change of HFD-*Mof*$^{+/-}$ versus *Mof*$^{+/+}$ and *Trim28*$^{+/D9}$ animals (original data from 16).
(h) MA-plot showing the DEG genes found in the transition of SD to HFD.

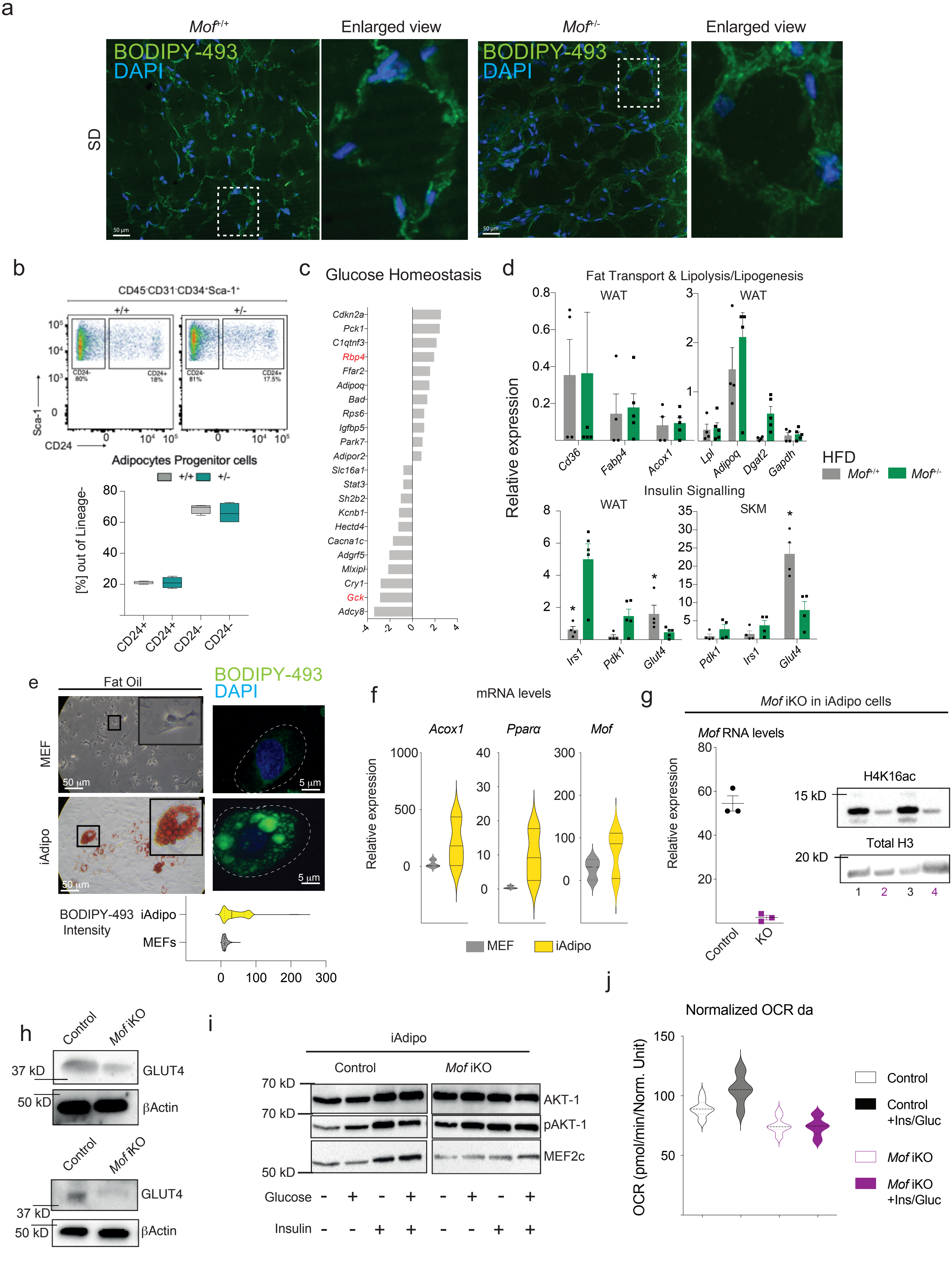

Supplementary Figure 6

Supplementary Fig. 6. MOF depletion is strongly associated with defects in glucose import transcription networks. Related to Fig. 5.

(a) Left: Immunofluorescence of white visceral adipocyte tissue after 26 weeks on SD. Nucleus depicted by DAPI staining (blue) and neutral lipid droplets stained by BODIPY-493 (green). Highlighted regions show the tecidual fat storage. Scale bar, 50 µm and enlarged view 5 µm. Right: Box-plots showing the number of adipocytes per field of view composed by 4 to 6 tiles, from 26-week-old *Mof*$^{+/+}$ (grey, *n*=5) and *Mof*$^{+/-}$ (teal, *n*=4) mice. *P*-value was calculated using the Mann-Whitney test.
(b) Adipogenic progenitor cell evaluation. Upper panel: Representative dot-plot showing the frequency of APC (CD45^{-}CD31^{-}CD34^{+}Sca1^{+}CD24$^{+/-}$) from *Mof*$^{+/+}$ and *Mof*$^{+/-}$ animals. Lower panel: Box-plot showing the frequency of CD24^{+} and CD24^{-} cells as a proportion of lineage-negative cells (Lin:CD45^{-}CD31^{-}) showing no significant difference for both APC populations. *n*=4 per genotype. *P*-values were calculated by two-sided *t*-test.
(c) Bar-Plots showing the WAT SD DEG Log_2 fold change associated with glucose homeostasis.
(d) RT-qPCR for genes related to lipolysis (*Acox1* and *Lpl*), fat transport (*Cd36* and *Fabp4*), lipogenesis (*Adipoq* and *Dgat2*) and insulin signaling (*Irs1, Pdk1* and *Glut4*) from HFD WAT. RT-qPCR for insulin signaling-related genes from HFD SKM. Expression was normalized to *Hprt*. Delta CT was normalized by the wild-type expression (*Mof*$^{+/+}$, *n*=4; *Mof*$^{+/-}$ *n*=5; number of animals). After the normality test, statistical analysis was performed by two-sided *t*-test, for parametric samples or two-sided Mann-Whitney test for non-parametric samples, **p*=0.05.
(e) Top: Representative bright-field of the fat-oil from mouse embryonic fibroblasts (MEFs) (left) and immunofluorescence showing neutral lipid staining (BODIPY-493, green) before and after in vitro adipocyte differentiation (right). Bottom: Quantification of the neutral lipid MFI is depicted by the violin-plots. Dotted lines show the quartiles and solid lines depict the medians. Undifferentiated cells are shown in black and iAdipo in yellow. *n*=4.
(f) RT-qPCR for *Acox-1, Mof* and *Ppara* before and after in vitro adipocyte differentiation. Expression was normalized to *Hprt*. Delta CT was normalized to untreated MEF expression (*n*=3). Dotted lines show the quartiled and dashed lines depict the medians.
(g) Left: Scatter-plot representing the RT-qPCR for *Mof* after 4-OHT treatment of iAdipo cells error bars represent ±SEM. Right: Immunoblot showing H4K16ac levels after *Mof* deletion. Total H3 was used as loading control. Each lane represents a biological replicate, *n*=3.
(h) Immunoblot showing GLUT4 protein levels for two independent iAdipocyte replicates. Actin was used as loading control.
(i) Immunoblot showing control and *Mof*-iKO iAdipo Ser473 phosphorylated AKT-1 and MEF2c levels at steady state, upon glucose, insulin or insulin-glucose challenges. The "+" sign indicates the treatment. Representative blot from 2 independent experiments showing similar results.
(j) Violin-plot showing the oxygen consumption rate (OCR) from *Mof*-iKO and control iAdipocytes. Empty bars represent steady state and filled bars after insulin and glucose (Ins/Gluc) treatment. Biological replicates (*n*=3). Dotted lines show the quartiles and dashed lines depict the medians.

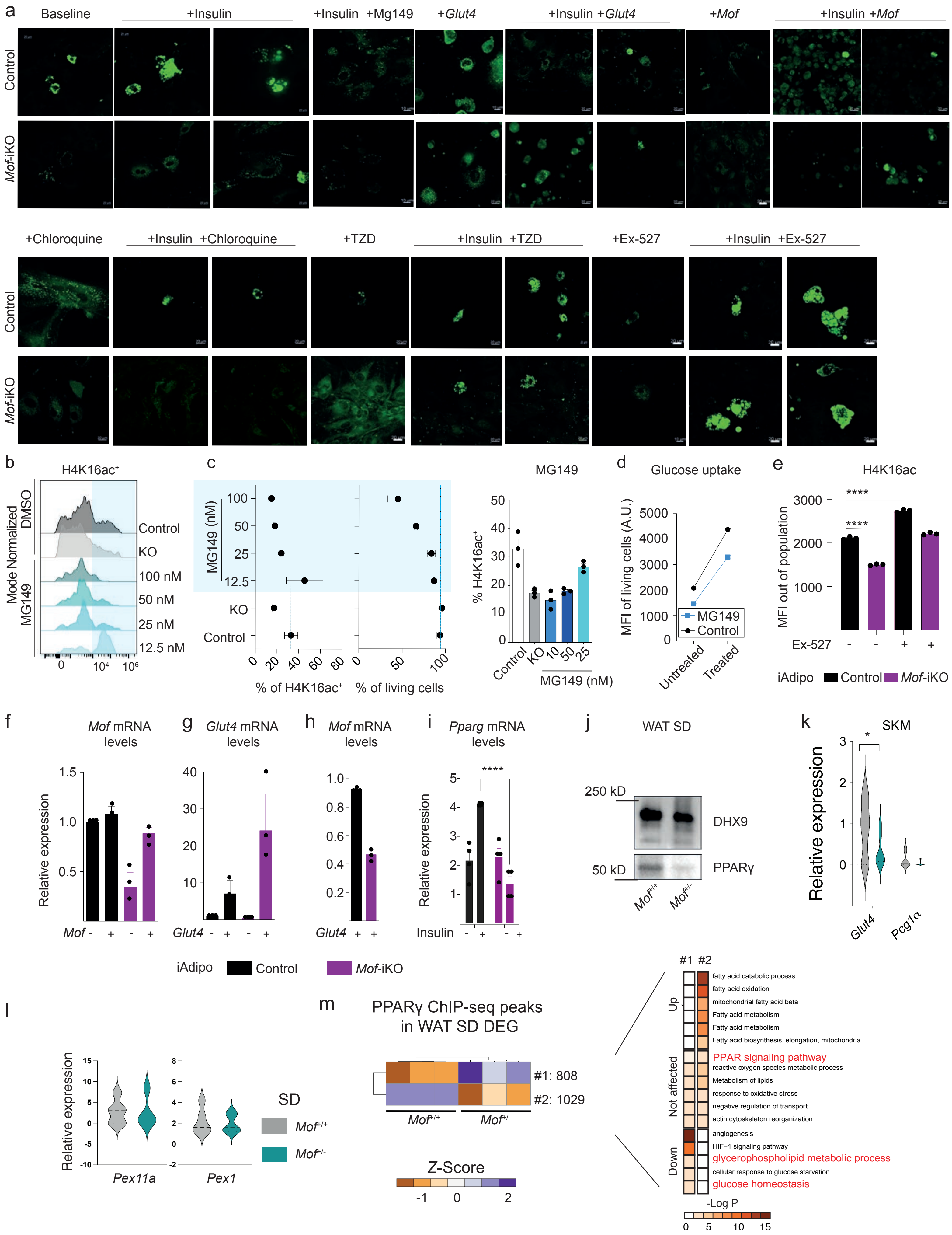

Supplementary Figure 7

Supplementary Fig. 7. Manipulation of the *Mof* and *Glut4* transcription network rescues fat storage in *Mof*-iKO adipocytes. Related to Fig. 6.

(a) Representative immunofluorescence showing the heterogeneity of droplets among the various iAdipo groups. Scale bar 20 µm. Data is representative of 3-5 independent biological replicates showing similar results.

(b) Representative histogram showing H4K16ac MFI. The blue box highlights the positive values based on the FMO control.

(c) Quantification of viable H4K16ac-positive cells (left and right plots) and percentage of living cells (middle plot) after different doses of MG149 treatment. Error bars represent means ± SEM (*n*=3).

(d) Line-plot showing the 2-NBDG import before and after insulin treatment in Control (iAdipo treated with DMSO) or iAdipo treated with MG149 (*n*=3).

(e) H4K16ac levels measured by immunofluorescence after Ex-527 treatment. Error bars represent means ±SEM (*n*=3). Statistical analysis was performed using the raw data and two-sided one-way ANOVA followed by Kruskal-Wallis comparison test ****p=0.000035.

(f-h) RT-qPCR for *Mof* after ectopic expression of wild type MOF. RT-qPCR for **(e)** *Glut4* or **(f)** *Mof* after wild type *Glut4* ectopic expression. Expression was normalized to *Hprt.* ΔCT was normalized by the wild-type expression (*n*=3). The "+" sign indicates ectopic expression. Error bars represent means ±SEM. Each dot represents an individual animal that cells were derived from, *n*=5.

(i) RT-qPCR for *Pparγ* in iAdipocytes, control shown in black and *Mof*-iKO in purple. The "+" sign indicates insulin treatment. Each dot represents an individual animal that cells were derived from, *n*=5. Statistical analysis performed by two-way ANOVA, followed by Holm-Sidak's comparison test, ****$p=10^{-16}$. Error bars represent means ±SEM.

(j) Representative immunoblot showing PPARγ levels in WAT from wild type and $Mof^{+/-}$ animals fed a SD. DHX9 was used as loading control. Representative blot from 2 independent experiments showing similar results.

(k) RT-qPCR for *Glut4* and *Pgc1α* in SKM. Statistical analysis performed by two-sided Mann-Whitney test, *p=0.02. Error bars represent means ±SEM. *n*=3.

(l) RT-qPCR for *Pex11a* and *Pex1*, both PPARγ targets not related to glucose homeostasis. Dotted lines show the quartiles and dashed lines depict the medians. *n*=3.

(m) Heatmap showing the Integration of PPARγ ChIP-seq peaks and DEG found in $Mof^{+/-}$ WAT SD (Left). Scale represents *Z*-Score. Right panel displays the associated KEGG pathway significantly enriched for genes that are bound by PPARg and up regulated, bound, but remain unaltered or bound and down regulated in $Mof^{+/-}$ WAT. Scale represents -Log(*p*-value). Benjamini-Hochberg method applied to calculate FDR adjusted.

Supplementary Table 1. Metabolic profiling of wild type and *Mof*$^{+/-}$ animals. Data was normalized using the quantile distribution on all raw data sets together using MetaboAnalyst Ver3. The fold change and log2 transformed values of the same, calculated on mean intensity values obtained after normalisation of each tissue sample set. *P*-Values are derived from two-sided unpaired t-tests performed without assuming consistent standard deviation.

Supplementary Table 2. Biomarkers identified in the *Mof*$^{+/-}$ samples. Biomarkers were identified using the Ingenuity Pathway Analysis (QIAGEN IPA) software. Statistical significance was obtained using the IPA in built false rate discovery (FDR).

Supplementary Table 3. Genomic transcriptional profile from wild type and *Mof*$^{+/-}$ WAT fed on SD or HFD. Statistical significance was conducted using the DESEQ2 software having two-sided FDR setted as 0.05.

Supplementary Table 4. MOF ChIP-seq characterization from wild type and *Mof*$^{+/-}$ WAT. The Benjamini-Hochberg method applied to calculate FDR adjusted *p*-values was used to define the enriched pathways.

Supplementary Table 5. Resources used in this study.
