# Supplementary material for: Histone H4 lysine 16 acetylation controls central carbon metabolism and diet-induced obesity in mice
Source: Nat Commun. 2021 Oct 27;12:6212. doi: 10.1038/s41467-021-26277-w (PMC8551339; doi:10.1038/s41467-021-26277-w)
Supplement: Supplementary file 7 — Supplementary Data 5 [file 41467_2021_26277_MOESM7_ESM.docx]

**Supplementary Data 5. Resources used in this study**

| REAGENT or RESOURCE | | SOURCE | | IDENTIFIER |
| --- | --- | --- | --- | --- |
| Antibodies | | | | |
| CD4 | | Biolegend | | 100511 |
| F4/80 | | Biolegend | | 123131 |
| bTCR | | Biolegend | | 109205 |
| IL10 | | BD | | 563276 |
| IL17A | | Biolegend | | 506911 |
| MOF | | Abcam | | ab72056 |
| H4K16ac | | Milipore | | 07-329 |
| Actin-HRP | | Santa Cruz | | sc-47778 |
| RBP3 | | Proteintech | | 14352-1-AP |
| H3 | | Milipore | | 04-928 |
| AKT-1 | | Cell Signalling | | 9272 |
| p-AKT1 | | Cell Signalling | | 9271 |
| GLUT4 | | Santa Cruz | | sc-53566 |
| MEF2C | | Cell Signalling | | 5030T |
| Insulin | | Invitrogen | | PA1-26938 |
| Oligonucleotides | | | | |
| *Pparγ ChIP* | | F-GTCTGAGGGACACGGGACCT  R-CTCAGCGGAGACCCAGTCAC | | |
| *Mef2c ChIP* | | F-TTTCCCATTCGTCTCCAGTC  R-GAGGAGGAAGGTGGAGGAAG | | |
| *Pcg1a ChIP* | | F-GTCTGAGGGACACGGGACCT  R-CTCAGCGGAGACCCAGTCAC | | |
| *Slc2a4 ChIP* | | F-AGACCCCGGCGTTCG  R-ATCCACTAAGGTTCCTCGCTC | | |
| *Cd36 cDNA* | | F-GGAGCCATCTTTGAGCCTTCA  R-GAACCAAACTGAGGAATGGATCT | | |
| *Il6 cDNA* | | F-CTGCAAGAGACTTCCATCCAG  R-AGTGGTATAGACAGGTCTGTTGG | | |
| *Adipoq cDNA* | | F-TGTTCCTCTTAATCCTGCCCA  R-CCAACCTGCACAAGTTCCCTT | | |
| *Fabp4 cDNA* | | F-ATCAGCGTAAATGGGGATTTGG  R-GTCTGCGGTGATTTCATCGAA | | |
| *Dgat2 cDNA* | | F-TTCCTGGCATAAGGCCCTATT  R-CCTCCAGACATCAGGTACTCG | | |
| *Plin2 cDNA* | | F-GACCTTGTGTCCTCCGCTTAT  CAACCGCAATTTGTGGCTC | | |
| *Lpl cDNA* | | F-TGGCGTAGCAGGAAGTCTGA  R-TGCCTCCATTGGGATAAATGTC | | |
| *Irs1 cDNA* | | F-CGATGGCTTCTCAGACGTG  R-CAGCCCGCTTGTTGATGTTG | | |
| *Pdk1 cDNA* | | F-AGGATCAGAAACCGGCACAAT  R-GTGCTGGTTGAGTAGCATTCTAA | | |
| *Slc2a4 cDNA* | | F-CCAGCCACGTTGCATTGTAG  R- GTGACTGGAAACACTGGTCCTA | | |
| *Hprt cDNA* | | F-TCAGTCAACGGGGGACATAAA  R-GGGGCTGTACTGCTTAACCAG | | |
| *NGN3 cDNA* | | F-TGGGGCAACTCCCAGGCGGG  R-TGGACAGTGGGCGCACCCGAGG | | |
| *CHOP* cDNA | | F-GGCCGGTTTCGAAGTTGATGCAAT  R-ACATCAACAGCAACAAGCCCGTAG | | |
| *iNOS* cDNA | | F-AATGTGGAGAAAGCCCCCTG  R-TGCATCCAGCTTGACCAGAG | | |
| *CAII* cDNA | | F-GCCTATAAAAGCTGGTGCCG  R-TACCCCCAGTGATGGGACAT | | |
| *CDH1* cDNA | | F-GGCGCTGGAGGGCCTGTCCTGA  R-CCCCTCACGGGGCAGCGGCA | | |
| *SNAI2* cDNA | | F-ACTCACACGGGGGAGAAGCCT  R-TCAGTGTGCTACACAGCAGCCA | | |
| Bacterial and Virus Strains | | | | |
| NEB® 5-alpha Competent *E. coli* | | New England BioLabs | | C2987I |
| Chemicals, Peptides, and Recombinant Proteins | | | | |
| Murine IL-2 | | PeproTech | | 212-12 |
| FGF10 | | PeproTech | | 100-26 |
| EGF | | Life Technologies | | PMG8043 |
| HGF | | PeproTech | | 100-39 |
| Gastrin I | | Sigma Aldrich | | G9145 |
| Y-27632 dihydrochloride | | Sigma Aldrich | | Y0503 |
| Noggin | | PeproTech | | 120-10C |
| *N*-acetylcysteine | | Sigma Aldrich | | A0737 |
| Nicotinamide | | Sigma Aldrich | | N0636 |
| B27 | | Life Technologies | | 12587-010 |
| N2 | | Life Technologies | | 17502048 |
| A 83-01 TGFβ inhibitor | | Tocris Bioscience | | 2939 |
| DAPT | | Sigma Aldrich | | D4902 |
| Brefeldin A | | BD | | 555029 |
| Foxp3 intracellular kit | | ebioscience | | 421403 |
| 4-Hydroxytamoxifen | | Sigma Aldrich | | H6278 |
| IBMX | | Sigma Aldrich | | [28822-58-4](https://www.sigmaaldrich.com/catalog/search?term=28822-58-4&interface=CAS%20No.&N=0&mode=partialmax&lang=de&region=DE&focus=product) |
| Troglitazone | | Tocris | | A3893 |
| Penicillin/Streptomycin | | Gibco | | 10378016 |
| TRIzol Reagent | | Thermo Fisher | | [15596018](http://www.thermofisher.com/order/catalog/product/15596018) |
| Superscript III Reverse Transcriptase | | Thermo Fisher | | 18080-093 |
| Superscript II Reverse Transcriptase | | Thermo Fisher | | 18064014 |
| RNAseOUT^TM^ | | Thermo Fisher | | 10777-019 |
| RNasin^®^ Ribonuclease inhibitor | | ProMega | | N2511 |
| Turbo DNase | | Life Technologies | | AM2238 |
| Dexamethasone | | Sigma Aldrich | | D4902 |
| FastStart SYBR mix | | Roche | | 04913850001 |
| PhosSTOP^TM^ | | Roche | | [PHOSS-RO](http://www.sigmaaldrich.com/ProductLookup.html?ProdNo=PHOSSRO&Brand=ROCHE) |
| cOmplete^™^ Protease Inhibitor Cocktail | | Roche | | 000000011697498001 |
| Avidin/Biotin Blocking Kit | | Biolegend | | SIG-31126 |
| Dynabeads™ Protein G for Immunoprecipitation | | Invitrogen | | 10004G |
| RNase A | | Qiagen | | 19101 |
| Histolemon-Erba | | Carlo-Erba | | [CH0274](http://www.bodanchimica.it/SCHEDA%20SICUREZZA/ItCH0274.htm) |
| Proteinase K | | Thermo Fisher | | EO0491 |
| GlycoBlue™ Coprecipitant | | Thermo Fisher | | AM9516 |
| Insulin-like growth factor 1 | | PeproTech | | 100-11 |
| BODIPY™ 493/505 | | Invitrogen | | D3922 |
| Mini RNA Isolation kit | | Zymo Research | | [R2071](https://www.zymoresearch.de/collections/direct-zol-rna-kits/products/direct-zol-rna-miniprep-plus-kits) |
| Plasmid DNA Midipreps Kits | | Zymo Research | | D4200-201 |
| MinElute PCR Purification | | Qiagen | | 28004 |
| Cvik1 | | NEB | | R0710S |
| Collagenase | | Sigma Aldrich | | C2674 |
| Dispase II | | Sigma Aldrich | | D4693 |
| Critical Commercial Assays | | | | |
| Seahorse Glycolysis set | | Agilent | | 103020-100 |
| Insulin ELISA | | Mercodia | | 10-1113-01 |
| Hba 1ac | | Cayman Chemicals | | 700540 |
| iDeal ChIP-Seq | | Diagenode | | C01010173 |
| Experimental Models: Cell Lines | | | | |
| PANC-1 | | BIOSS Freiburg | | CVCL_0480 |
| L-Wnt3a expressing mouse friboblasts | | ATCC | | CRL-2647 |
| 293T-HA-Rspo1-Fc cell line | | Millipore | | SCC111 |
| Experimental Models | | | | |
| FVB-Tg (Ckm-cre) 5Khn/J | | The Jackson Laboratory | | RRID:IMSR_JAX:004682 |
| *Mof* heterozygous | | Tim Thomas | |  |
| Software and Algorithms | | | | |
| Prism 6 | | GraphPad Software | | http://www.graphpad.com/scientific- software/prism/ |
| R | | 3.6.2 | | https://www.rstudio.com |
| SnakePipes | | 2.1.2 | | https://snakepipes.readthedocs.io/en/latest/ |
| Metascape | | http://metascape.org/gp/index.html | | (Tripathi et al., 2015) |
| DESeq2 | | 1.16.1 | | (Love et al., 2014) |
| ggplot2 | | 2.2.1 | | https://cran.r-project.org/web/packages/ggplot2/index.html |
| pheatmap | | 1.0.8 | | https://cran.r-project.org/web/packages/pheatmap/pheatmap.pdf |
| matrixStats | | 0.52.2 | | https://cran.rstudio.com/web/packages/matrixStats/index.html |
| Biobase | | 2.36.2 | | http://bioconductor.org/packages/release/bioc/html/Biobase.html |
| GenomeInfoDb | | 1.28.3 | | https://bioconductor.org/packages/release/bioc/html/GenomeInfoDb.html |
| GenomeInfoDb | | 1.12.2 | | https://bioconductor.org/packages/release/bioc/html/GenomeInfoDb.html |
| IRanges | | 2.10.2 | | http://bioconductor.org/packages/release/bioc/html/IRanges.html |
| BiocGenerics | | 0.22.0 | | http://bioconductor.org/packages/release/bioc/html/BiocGenerics.html |
| RColorBrewer | | 1.1-2 | | https://cran.r-project.org/web/packages/RColorBrewer/RColorBrewer.pdf |
| Galaxy Platform | |  | | [^134^](https://paperpile.com/c/JdXuGi/0f9tk) |
| Deeptools | | Galaxy Version 3.5.0 | | [^130^](https://paperpile.com/c/JdXuGi/L4eXP) |
| ChipAnno | | 3.12.0 | | [^135^](https://paperpile.com/c/JdXuGi/UvWjg) |
| ChiPseeker | | 1.14.0 | | [^131^](https://paperpile.com/c/JdXuGi/oxEJt) |
| ClusterProfiler | | 3.11.0 | | [^136^](https://paperpile.com/c/JdXuGi/Jsd6c) |
| Genie3 | | 3.11.0 | | [^56^](https://paperpile.com/c/JdXuGi/jittD) |
| Genome-wide datasets | | | | |
| Fat scRNA-seq | SRA653146//SRS2874271 | | PangloDB | |
| Heart scRNA-seq | SRA762414//SRS3703557 | | PangloDB | |
| Pancreas scRNA-seq | SRA745567//SRS3600296 | | PangloDB | |
| Kidney scRNA-seq | SRA635314//SRS2727269. | | PangloDB | |
| SKM scRNA-seq | SRA653146//SRS2874279 | | PangloDB | |
| Liver scRNA-seq | [SRA739096](https://www.ncbi.nlm.nih.gov/sra/?term=SRA739096)/SRS3545822 | | PangloDB | |
| *Trim28*^+/D9^ WAT RNA-seq | PRJEB11740 | | [^76^](https://paperpile.com/c/JdXuGi/P38TK) | |
| PPARγ ChIP-seq | GSM4634568 | | SRX8605838 | |
| *Mof^+/-^* WAT RNA-seq | GSE156463 | | This paper | |
| *Mof^+/-^* HFD WAT RNA-seq | GSE156463 | | This paper | |
| *Mof*^+/+^ WAT MOF ChIP-seq | GSE156463 | | This paper | |
| *Mof^+/-^* WAT MOF ChIP-seq | GSE156463 | | This paper | |
