# Supplementary material for: Histone H4 lysine 16 acetylation controls central carbon metabolism and diet-induced obesity in mice
Source: Nat Commun. 2021 Oct 27;12:6212. doi: 10.1038/s41467-021-26277-w (PMC8551339; doi:10.1038/s41467-021-26277-w)
Supplement: Supplementary file 8 — Reporting Summary [file 41467_2021_26277_MOESM8_ESM.pdf]

nature portfolio

Corresponding author(s): Asifa Akhtar

Last updated by author(s): 23/8/21

Reporting Summary

Nature Portfolio wishes to improve the reproducibility of the work that we publish. This form provides structure for consistency and transparency in reporting. For further information on Nature Portfolio policies, see our Editorial Policies and the Editorial Policy Checklist.

Statistics

For all statistical analyses, confirm that the following items are present in the figure legend, table legend, main text, or Methods section.

n/a	Confirmed	
☐	☒	The exact sample size (*n*) for each experimental group/condition, given as a discrete number and unit of measurement
☐	☒	A statement on whether measurements were taken from distinct samples or whether the same sample was measured repeatedly
☐	☒	The statistical test(s) used AND whether they are one- or two-sided<br>*Only common tests should be described solely by name; describe more complex techniques in the Methods section.*
☐	☒	A description of all covariates tested
☐	☒	A description of any assumptions or corrections, such as tests of normality and adjustment for multiple comparisons
☐	☒	A full description of the statistical parameters including central tendency (e.g. means) or other basic estimates (e.g. regression coefficient) AND variation (e.g. standard deviation) or associated estimates of uncertainty (e.g. confidence intervals)
☐	☒	For null hypothesis testing, the test statistic (e.g. *F*, *t*, *r*) with confidence intervals, effect sizes, degrees of freedom and *P* value noted<br>*Give P values as exact values whenever suitable.*
☒	☐	For Bayesian analysis, information on the choice of priors and Markov chain Monte Carlo settings
☒	☐	For hierarchical and complex designs, identification of the appropriate level for tests and full reporting of outcomes
☐	☒	Estimates of effect sizes (e.g. Cohen's *d*, Pearson's *r*), indicating how they were calculated

Our web collection on statistics for biologists contains articles on many of the points above.

Software and code

Policy information about availability of computer code

Data collection	no software was used for data collection
Data analysis	Omu R package version 1.0.4<br>MetaboAnalyst software version 3.0<br>SnakePipes version 2.1.2<br>DESEQ2 version 1.26<br>Seurat version 3.0<br>GENIE3 Bioconductor version 3.11<br>clusterProfiler Bioconductor version 3.13<br>pheatmap version 1.0.8<br>MACS2 Galaxy version 2.1.1.20160309.3<br>BEDtools sort Galaxy version 2.27.0.0<br>BamCompare Galaxy version 2.5.1.0.0<br>DeepTools2 version 3.5.0<br>ShinyGo version 0.61<br>FlowJo version 10<br>Graphpad Prism version 6<br>R version 3.6.2

For manuscripts utilizing custom algorithms or software that are central to the research but not yet described in published literature, software must be made available to editors and reviewers. We strongly encourage code deposition in a community repository (e.g. GitHub). See the Nature Portfolio guidelines for submitting code & software for further information.

Data

Policy information about availability of data

All manuscripts must include a data availability statement. This statement should provide the following information, where applicable:

- Accession codes, unique identifiers, or web links for publicly available datasets
- A description of any restrictions on data availability
- For clinical datasets or third party data, please ensure that the statement adheres to our policy

Single-cell RNA seq data were obtained from the PagloDB database:
Heart:SRA762414/SRS3703557
Fat:SRA653146/SRS2874271
Pancreas: SRA745567/SRS3600296
Kidney:SRA635314/SRS2727269
SKM:SRA653146/SRS2874279
Liver: SRA739096/SRS3545822

Bulk RNA-seq data were obtained from GEO:
Trim28+/D9: PRJEB11740
Mof+/- and +/+ WAT RNA-seq: GSE156463

ChIPseq data were obtained from GEO:
PPARγ ChIP-seq: GSM4634568
Mof +/- and +/+ WAT MOF ChIP-seq: GSE156463

Field-specific reporting

Please select the one below that is the best fit for your research. If you are not sure, read the appropriate sections before making your selection.

☒ Life sciences ☐ Behavioural & social sciences ☐ Ecological, evolutionary & environmental sciences

For a reference copy of the document with all sections, see nature.com/documents/nr-reporting-summary-flat.pdf

Life sciences study design

All studies must disclose on these points even when the disclosure is negative.

Sample size	The sample size is describe in every figure as well as the appropriated statistical test. Wherein first the samples were evaluated for their normality distribution using Shapiro-Wilk test and the Agostin test. If normal distribution was scored the statistical analysis was conducted using a normal distribution test e,g: one-way ANOVA followed by Tukey or Student t-test. If the sample distribution was not normal the follow analysis was conducted by Mann-Whitney test or one-way ANOVA followed by Kruskal-Wallis test. Confidence value was established as 95% and therefore p-value as 0.05. Longitudinal data were analyzed using the two-way ANOVA test. Correlations were scored by the Pearson Correlation.
Data exclusions	No data were excluded from the analyses
Replication	Every in vitro experiment were performed at least 3 times as independent experiments. Longitudinal In vivo data were generated using at least a n equal to 4-5 for both gender and measurements taken at least 2 times. All the attempts at replication were successful.
Randomization	Experimental groups were allocated based on their genotypes. For the longitudinal in vivo experiments we grouped 7 weeks old animals both genders (minimum of 4 animals per condition) from both genotypes. For punctual we used adults littermate animals, 8-10 weeks old. Overall, samples were allocated based on their genotype and treatments.
Blinding	Blinding was not applicable to this study. Since differential treatment or assessment of participants did not cause potential bias and it is not a trial study.

Reporting for specific materials, systems and methods

We require information from authors about some types of materials, experimental systems and methods used in many studies. Here, indicate whether each material, system or method listed is relevant to your study. If you are not sure if a list item applies to your research, read the appropriate section before selecting a response.

Materials & experimental systems

n/a	Involved in the study
☐	☒ Antibodies
☐	☒ Eukaryotic cell lines
☒	☐ Palaeontology and archaeology
☐	☒ Animals and other organisms
☒	☐ Human research participants
☒	☐ Clinical data
☒	☐ Dual use research of concern

Methods

n/a	Involved in the study
☐	☒ ChIP-seq
☐	☒ Flow cytometry
☒	☐ MRI-based neuroimaging

Antibodies

Antibodies used

CD3, Biolegend, cat. number: 100217, PERCP-Cy5.5, clone: 17A2. Dilution: 1:400
CD4, Biolegend, cat.number: 100446, A700, clone:GK1.5. Dilution: 1:400
CD8a, Biolegend, cat. number:100707, PE, clone: 53-6.7.Dilution: 1:800
IFN-gamma, Biolegend, cat.number: 505805, FITC, clone: XMG1.2. Dilution: 1:200
IL-17A, Biolegend , cat. number: 506911, A647, clone: TC11-18H10.1. Dilution: 1:200
RORgammat, BD, cat.number: 562607, PERCP-Cy5.5, clone: Q31-378. Dilution: 1:400
FoxP3, Biolegend, cat.number: 126421, AF700, clone: MF-14. Dilution: 1:400
IL-4, Biolegend, cat.number: 504119, BV421, clone: 11B11. Dilution: 1:400
IL-10, BD, cat.number: 563276, BV421,clone: JES5-16E3. Dilution: 1:400
F4/80, Biolegend, cat.number: 123131, BV421, clone: BM8.Dilution: 1:800
bTCR , Biolegend ,cat. number: 109205, FITC, clone: H57-597. Dilution: 1:400
MOF, Abcam, cat.number: ab72056, Dilution: 1:1000
MOF, Millipore, cat. number:ABE479. Dilution: 1:2000
H4K16ac, Millipore, cat.number: 07-329.Dilution: 1:2000
Actin-HRP, Santa Cruz,cat.number: sc-47778.Dilution: 1:10000
RBP3, Proteintech, cat. number: 14352-1-AP.Dilution: 1:10000
H3, Milipore, cat. number: 04-928.Dilution: 1:10000
AKT-1 ,Cell Signalling, cat.number: 9272.1:2000
p-AKT1, Cell Signalling, cat.number: 9271.1:2000
GLUT4, Santa Cruz, cat.number: sc-53566.1:500
MEF2C, Cell Signalling, cat.number: 5030T.1:2000
Insulin, Invitrogen, cat. number: PA1-26938.1:500

Validation

MOF and H4K16ac:
Tested on Mof KO lysates. Expected band lost in Mof and H4K16ac KO samples via Western blot.

GLUT4, MEF2C, AKI-1, pAKT1
Tested on WAT cells treated with insulin. Expected bands increase in the treated samples via Western blot.

Insulin
Were validated by western blot comparing pancreas samples versus MEF
.CD3, CD4,CD8a,IFN-g,IL17A, RORgt, FoxP3, IL-4,IL-10,F4/80,bTCR were validated and titrated by FACS using T cells.

Eukaryotic cell lines

Policy information about cell lines

Cell line source(s)

PANC-1, BIOSS Freiburg, CVCL_0480
L-Wnt3aexpressing mouse fibroblast, ATCC, CRL-2647
293T-HA-Rspo1-Fc cell line, Millipore SCC111

Authentication

Cell lines were not authenticated

Mycoplasma contamination

Cells were negative for Micoplasma contamination

Commonly misidentified lines
(See ICLAC register)

No commonly misidentified cell lines were used in the study

Animals and other organisms

Policy information about studies involving animals; ARRIVE guidelines recommended for reporting animal research

Laboratory animals	Animals were maintained on a 14/10-hour light-dark cycle under stable humidity and temperature. For standart diet experiments the animals were provided with a normal chow diet 15% fat (Ssniff GmbH), while obese animals were fat with high fat diet (60% kcal% fat, Reasearch Diet). Both groups were fed ad libitum, had free access to water and refinement methods as nesting material were employed. The gain of total body mass was recorded every second week. Every mouse stain in this study was back crossed with C57BL/6J mice. All animals procedures are in agreement with and approved by the responsible Welfare Committes (Regierungsrasidium Freiburg, Karlsruhe/ Germany, license, G17/150), confirming to the Guide for the Care and Use of laboratory Animals published by the National Institute of Health (publication No. 85-23, revised 1996).<br>Mof floxed and heterozygous (Thomas et al., 2008)<br>Caag-Cre-ERT2 (from Jackson lab).<br>Experimentations were undertaken on adult (8-10 week olds) males and females.
Wild animals	no wild animals were used in the study
Field-collected samples	no field collected samples were used in the study.
Ethics oversight	All animals procedures are in agreement with and approved by the responsible Welfare Committes (Regierungsrasidium Freiburg, Karlsruhe/ Germany, license, G17/150)

Note that full information on the approval of the study protocol must also be provided in the manuscript.

ChIP-seq

Data deposition

☒ Confirm that both raw and final processed data have been deposited in a public database such as GEO.

☒ Confirm that you have deposited or provided access to graph files (e.g. BED files) for the called peaks.

Data access links
May remain private before publication.

GSE156463

Files in database submission

GSE154967 MOF haploinsufficiency triggers diet-induced obesity resistance (RNA-seq) Jul 23, 2021 approved None
GSM4685051 SD_control1 Jul 23, 2021 approved TXT
GSM4685052 SD_control2 Jul 23, 2021 approved TXT
GSM4685053 SD_control3 Jul 23, 2021 approved TXT
GSM4685054 SD_het1 Jul 23, 2021 approved TXT
GSM4685055 SD_het2 Jul 23, 2021 approved TXT
GSM4685056 SD_het3 Jul 23, 2021 approved TXT
GSM4685057 HFD_control1 Jul 23, 2021 approved TXT
GSM4685058 HFD_control2 Jul 23, 2021 approved TXT
GSM4685059 HFD_het1 Jul 23, 2021 approved TXT
GSM4685060 HFD_het2 Jul 23, 2021 approved TXT

GSE156462 MOF haploinsufficiency triggers diet-induced obesity resistance (ChIP-seq) Jul 23, 2021 approved BIGWIG BIGWIG BIGWIG BIGWIG BED BED
GSM4732018 20L004198: WT_Mof_ChIP_Rep1 Jul 23, 2021 approved None
GSM4732019 20L004199: WT_Mof_ChIP_Rep2 Jul 23, 2021 approved None
GSM4732020 20L004200: Het_Mof_ChIP_Rep1 Jul 23, 2021 approved None
GSM4732021 20L004201: Het_Mof_ChIP_Rep2 Jul 23, 2021 approved None
GSM4732022 20L004202: WT_H3_ChIP_Rep1 Jul 23, 2021 approved None
GSM4732023 20L004203: WT_H3_ChIP_Rep2 Jul 23, 2021 approved None
GSM4732024 20L004204: Het_H3_ChIP_Rep1 Jul 23, 2021 approved None
GSM4732025 20L004205: Het_H3_ChIP_Rep2 Jul 23, 2021 approved None
GSM4732026 20L004206: WT_Input_Rep1 Jul 23, 2021 approved None
GSM4732027 20L004207: WT_Input_Rep2 Jul 23, 2021 approved None
GSM4732028 20L004208: Het_Input_Rep1 Jul 23, 2021 approved None
GSM4732029 20L004209: Het_Input_Rep2 Jul 23, 2021 approved None

Genome browser session
(e.g. UCSC)

No longer applicable. Browser session was conducted locally using IGV.

Methodology

Replicates	For ChIP-seq we used 2 littermates from each genotype

Sequencing depth

Libraries were prepared using the NEVNext Ultra II DNA Librabry Prep Kit for Illumina according to manufactures's instructions (NEB, cat. Number: E7645). ChIP librabries were sequenced with 2x 50bp paired-ends reads on illumina NovaSeq6000 sequencer

Antibodies

MOF, Abcam, cat.number: ab72056.
MOF, Millipore, cat. number:ABE479.
H3, Milipore, cat. number: 04-928.

Peak calling parameters

ChIP-seq datasets were mapped to GRCm38/Mm10 with default paired-end parameters from snakePipes version 2.1.2. Peaks were called with MACS2 (Galaxy version 2.1.1.20160309.3), and bandwidth was set to 200, lower mfold bound to 5, upper mfold bound to 500, and the q-value to 0.1. Input was used as control to call peaks. Peaks from each cell type were merged using cat, BEDtools sort (Galaxy version 2.27.0.0) and piped to BEDtools merge with a distance of 1000 bp. BamCompare (Galaxy version 2.5.1.0.0) with a bin size of 50 bp. The data were normalized as Log2 fold change over H3 immunoprecipitation. For plotting heatmaps were generated using deepTools2 compute matrix and plotHeatmap function from DeepTools2 version 3.5.0 113. Box-plots were generated using DeepTools2 multiBigwigSummary scores. The Bioconductor package ChIP-Seeker 114. was used to retrieve the nearest genes around the peak, annotate the genomic region of the peak, and peak features annotation. GO term analysis was performed using the Metascape platform and the ShinyGo v0.61 database.

Data quality

Initial QC were ensured by deeptools FASTQC. Sample correlation were scored using the plotCorrelation function, and Pearson test used to evaluated replicates correlation. For peak calling peaks were called with MACS, and banwidth was set to 200, lower mfold bound to 5, upper mfold bound to 500, and the q-value to 0.1. Input was used as control to call peaks. Peaks from each cell type were merged using cat, BEDtools and piped to BEDtools merge with a distance of 1000bp. BamCompare with bin size of 50 bp. The data were normalized as Log2 fols change over H3 immunoprecipitation. For plotting heatmaps were generated using deepTools2 compute matrix and plotHeatmap function from DeepTools2 version 3.5.0. To further validated our ChI peak we integrated our RNAseq profile to validated a decrease in gene expression.

Software

SnakePipes version 2.1.2
DESEQ2 version 1.26
pheatmap version 1.0.8
MACS2 Galaxy version 2.1.1.20160309.3
BEDtools sort Galaxy version 2.27.0.0
BamCompare Galaxy version 2.5.1.0.0
DeepTools2 version 3.5.0
Graphpad Prism version 6
ChIP-seeker Bioconductor version: Release (3.13)
R version 3.6.2

Flow Cytometry

Plots

Confirm that:

☒ The axis labels state the marker and fluorochrome used (e.g. CD4-FITC).

☒ The axis scales are clearly visible. Include numbers along axes only for bottom left plot of group (a 'group' is an analysis of identical markers).

☒ All plots are contour plots with outliers or pseudocolor plots.

☒ A numerical value for number of cells or percentage (with statistics) is provided.

Methodology

Sample preparation

Mof knockdown in in vitro differentiated pancreatic islets and insulin response
The siRNA knockdowns (5 nM of Silencer Select (Ambion) control siRNA 4390846 or siRNA s38569 for MOF were performed on in vitro differentiated pancreatic islets for 3 days in triplicates using RNAiMAX (Thermo Fisher Scientific). Before knockdown, cells were seeded on 12-well plates in which in the bottom of the well we insert a coverslip. After, silencing cells were then crosslinked with 4 % methanol-free formaldehyde in PBS at room temperature for 10 min and permeabilized with 0.1 % Triton-X and 1 % BSA in PBS for 30 min at room temperature. Primary antibodies against insulin were diluted (1:100) in FACS-buffer and incubated at 4 °C for ~16 h. After, wells were washed 2x with PBS-T and secondary fluorescently labelled antibodies were used to reveal target proteins. DAPI was used to stain DNA. Imaging was performed with the LSM780 confocal microscope.

Mof and Glut4 in-vitro differentiated adipocytes
The Glut4 coding sequence was sub-cloned from Addgene plasmid number 52872 and Mof coding sequence were cloned into a pcDNATM5/pCMV vector. In vitro differentiated adipocytes were transfected with this vector by LTX Lipofectamine (ThermoFisher, #A12621) with a 5:1 lipid [Lipofectamine LTX (microliters)]–to–DNA (micrograms) ratio. Twelve hours after transfection, the media was changed to the respective cell culture media. Functional experiments were conducted 24 hours after the first transfection. Transfection efficiency was conducted by pmaxGFP Vector (Lonza, V4XP-3012).

Glucose uptake assay

For measurement of glucose uptake, at least 2000 wild-type and Mof-iKO iAdipo cells were cultured in DMEM-10% supplemented with 10% glucose with or without 10 g/ml of insulin with 200 mM of -(n-(7-nitrobenz-2-oxa-1,3-diazol-4-ylamino)-2-deoxyglucose (2-NBD-Glucose, 2 NBDG) for 30 min. Then cells were washed 2x with PBS and resuspended in

FACS-Buffer (0.1%BSA, 1mM EDTA, PBS) containing DAPI and immediately analyzed by flow cytometry using the BD cytometer Fortessa I/II. The data was then analyzed using the FlowJo v.10 software.

Livers from 26-week-old HFD-treated Mof+/+ and Mof+/- animals were harvested and placed in cold PBS. Ductal liver organoids were generated according to published protocol 94 with modifications. In brief, livers were minced thoroughly and washed 3 times in cold wash buffer (GlutaMAX, Gibco #35050-038, 1% FCS, 100 U/ml penicillin and 100 μg/ml streptomycin (Gibco #15140-122)) to remove fat. Tissue was digested in pre-warmed digestion media (wash buffer, 0.25mg/ml Collagenase (Sigma Aldrich #C2674), 0.125mg/ml (Life Technologies #17105-041) at 37°C on a rotating wheel for 1 hour. Tissue was pelleted, supernatant discarded and 1 hour digestion was repeated using fresh digestion buffer. Ductal structures were pelleted and washed three times in cold wash buffer. Ductal fragments were handpicked and seeded into Matrigel® (Corning, #356231) domes (50 fragments per 50ul of Matrigel®). Upon solidification of the basement matrix, domes were overlaid with isolation medium and incubated for 3 days under standard cell culturing conditions (37°C with 5 % CO2) after which media was exchanged to expansion media. Ductal organoids were maintained and 14 day hepatocyte differentiation protocol was performed as described elsewhere.

Liver organoid and CD4+ T cell co-culture
Fluorescence-activated cell sorting
For co-culture, wild-type thymus were isolated and thymocytes retrieve by mechanical filtering on a 70 μm cell strainer. The cells were resuspended in PBS, centrifuged at 500g for 5 min and the pellet was resuspended in ACK lysing buffer for erythrocyte cleaning. After 10 mins, PBS was added to the cellular suspension followed by centrifugation. The remaining leukocytes where resuspended in FACS-buffer containing zombie-dye for dead cells, fluorophore-conjugated primary antibodies against CD3 (PERCP-Cy5.5, clone:), CD4 (A700, clone) and CD8a (PE, clone) and kept on ice for 30 min. After, cells were resuspended in a 10x v/v FACS buffer and centrifuged at 10.000 rpm for 30 seconds. The pellet was resuspended in FACS buffer and naive CD8a+ cells sorted using the BD Aria-FACS Fusion II using a nozzle of 70 microm and maximum flow of 15.000 events/second rate. A 95% pure population was sorted and used for co-culture.
Organoids and naive Th cell co-culture
Sorted purified naive T cells were then co-culture with liver organoids following a 1:2000 proportion in the presence of IL-2 (10 ng/mL, Peprotech 212-12). T cells were further separated into two groups a) stimulated with CD3:CD28 dyna-beads (Thermo Fisher, 11456D) in a 1:1 ratio or b) no stuli. 5 days prior to the co-culture experiment differentiated hepatic organoids were cultured in AIM V™ media (Thermo Fisher) supplemented with 3 μM Dexamethasone. After 5 days of co-culture cells were harvested and prepared for flow cytometry analysis.
For cytokine flow cytometry evaluation we added 1X Brefeldin-A for at least 10 hrs prior to analysis. The cells were then transferred to 1.5 ml tubes and centrifuged at 10.000 rpm for 30 seconds. The remaining pellet was washed 2x with FACS-buffer and fluorophore-conjugated primary antibodies against CD4 (PE, Biolegend 100511, 1:400) and viability dye (zombie-dye aqua or green) was added and incubated for 30 min incubation on ice. Then the sample was centrifuged and pellet washed twice with 1x wash-buffer (Foxp3/Transcription Factor Staining Buffer Set, Affymetrix, eBioscience, USA, #00-5523-00), followed by 40 min incubation on ice with the "Perm/Fix" solution (1:4 dilution) (Foxp3/Transcription Factor Staining Buffer Set, Affy- metrix, eBioscience, USA, #00-5523-00). Samples were washed once with 1x wash buffer and twice with FACS-buffer followed by incubation with fluorophore-conjugated primary antibodies against IFN-gamma (FITC, clone), IL-17A (A647, clone), RORgammat (PERCP-Cy5.5, clone), FoxP3 (AF700, clone) and/or IL-4 (BV421, clone), IL-10(BV421,clone) as indicated in the Figures. The cells were incubated for ~16 hrs, washed twice with FACS-buffer and immediately acquired using the BD Fortessa II or Fortessa I cytometer. Data was next analyzed using the FlowJo v.10 software.
Livers from 26-week-old HFD-treated Mof+/+ and Mof+/- animals were harvested and placed in cold PBS. Ductal liver organoids were generated according to published protocol 94 with modifications. In brief, livers were minced thoroughly and washed 3 times in cold wash buffer (GlutaMAX, Gibco #35050-038, 1% FCS, 100 U/ml penicillin and 100 μg/ml streptomycin (Gibco #15140-122)) to remove fat. Tissue was digested in pre-warmed digestion media (wash buffer, 0.25mg/ml Collagenase (Sigma Aldrich #C2674), 0.125mg/ml (Life Technologies #17105-041) at 37°C on a rotating wheel for 1 hour. Tissue was pelleted, supernatant discarded and 1 hour digestion was repeated using fresh digestion buffer. Ductal structures were pelleted and washed three times in cold wash buffer. Ductal fragments were handpicked and seeded into Matrigel® (Corning, #356231) domes (50 fragments per 50ul of Matrigel®). Upon solidification of the basement matrix, domes were overlaid with isolation medium and incubated for 3 days under standard cell culturing conditions (37°C with 5 % CO2) after which media was exchanged to expansion media 94. Ductal organoids were maintained and 14 day hepatocyte differentiation protocol was performed as described in 94.

Liver organoid and CD4+ T cell co-culture
Fluorescence-activated cell sorting
For co-culture, wild-type thymus were isolated and thymocytes retrieve by mechanical filtering on a 70 μm cell strainer. The cells were resuspended in PBS, centrifuged at 500g for 5 min and the pellet was resuspended in ACK lysing buffer for erythrocyte cleaning. After 10 mins, PBS was added to the cellular suspension followed by centrifugation. The remaining leukocytes where resuspended in FACS-buffer containing zombie-dye for dead cells, fluorophore-conjugated primary antibodies against CD3 (PERCP-Cy5.5, clone:), CD4 (A700, clone) and CD8a (PE, clone) and kept on ice for 30 min. After, cells were resuspended in a 10x v/v FACS buffer and centrifuged at 10.000 rpm for 30 seconds. The pellet was resuspended in FACS buffer and naive CD8a+ cells sorted using the BD Aria-FACS Fusion II using a nozzle of 70 microm and maximum flow of 15.000 events/second rate. A 95% pure population was sorted and used for co-culture.
Organoids and naive Th cell co-culture
Sorted purified naive T cells were then co-culture with liver organoids following a 1:2000 proportion in the presence of IL-2 (10 ng/mL, Peprotech 212-12). T cells were further separated into two groups a) stimulated with CD3:CD28 dyna-beads (Thermo Fisher, 11456D) in a 1:1 ratio or b) no stimuli. 5 days prior to the co-culture experiment differentiated hepatic organoids were cultured in AIM V™ media (Thermo Fisher) supplemented with 3 μM Dexamethasone. After 5 days of co-culture cells were harvested and prepared for flow cytometry analysis.
For cytokine flow cytometry evaluation we added 1X Brefeldin-A for at least 10 hrs prior to analysis. The cells were then transferred to 1.5 ml tubes and centrifuged at 10.000 rpm for 30 seconds. The remaining pellet was washed 2x with FACS-buffer and fluorophore-conjugated primary antibodies against CD4 (PE, Biolegend 100511, 1:400) and viability dye (zombie-dye aqua or green) was added and incubated for 30 min incubation on ice. Then the sample was centrifuged and pellet washed twice with 1x wash-buffer (Foxp3/Transcription Factor Staining Buffer Set, Affymetrix, eBioscience, USA,

#00-5523-00), followed by 40 min incubation on ice with the "Perm/Fix" solution (1:4 dilution) (Foxp3/Transcription Factor Staining Buffer Set, Affy- metrix, eBioscience, USA, #00-5523-00). Samples were washed once with 1x wash buffer and twice with FACS-buffer followed by incubation with fluorophore-conjugated primary antibodies against IFN-gamma (FITC, clone), IL-17A (A647, clone), RORgammat (PERCP-Cy5.5, clone), FoxP3 (AF700, clone) and/or IL-4 (BV421, clone), IL-10(BV421,clone) as indicated in the Figures. The cells were incubated for ~16 hrs, washed twice with FACS-buffer and immediately acquired using the BD Fortessa II or Fortessa I cytometer. Data was next analyzed using the FlowJo v.10 software.

Instrument	BD cytometer Fortessa II for analytical experiments and ARia Fusion II for sorting experiments
Software	BD Viva (version 9.0) and FlowJo (version 10)
Cell population abundance	A minimum of 95% pure population was sorted and used for co-culture.
Gating strategy	Single cells were collected based on FSC/SSC parameters:<br>FSC-Area versus FSC-height -> FSC-height versus FSC-width -> SSC-area versus SSC-height -> FSC-area versus SSC-area. Viable cells were gated using the far red/green or UV Zombie-dye.<br>Glucose up-taken was analysed from singlets and living iAdipo.<br>Primary pancreas: Singlets, living and Insulin positive cells.<br>Th1 cells: CD3+-CD4+ CD8-IFNg+<br>Th2 cells: CD3+-CD4+ CD8-IFNg-IL4+<br>Treg: Th1 cells: CD3+-CD4+ CD8-Foxp3+Il-10+<br>Th17 cells: CD3+-CD4+ CD8-IL17+RORgt+<br>Progenitor Adipocytes: CD45-CD31-CD34+Sca-1+CD24+

☒ Tick this box to confirm that a figure exemplifying the gating strategy is provided in the Supplementary Information.
