# Supplementary material for: Histone H4 lysine 16 acetylation controls central carbon metabolism and diet-induced obesity in mice
Source: Nat Commun. 2021 Oct 27;12:6212. doi: 10.1038/s41467-021-26277-w (PMC8551339; doi:10.1038/s41467-021-26277-w)

FACS Gating Strategies

Related to Figure 5j

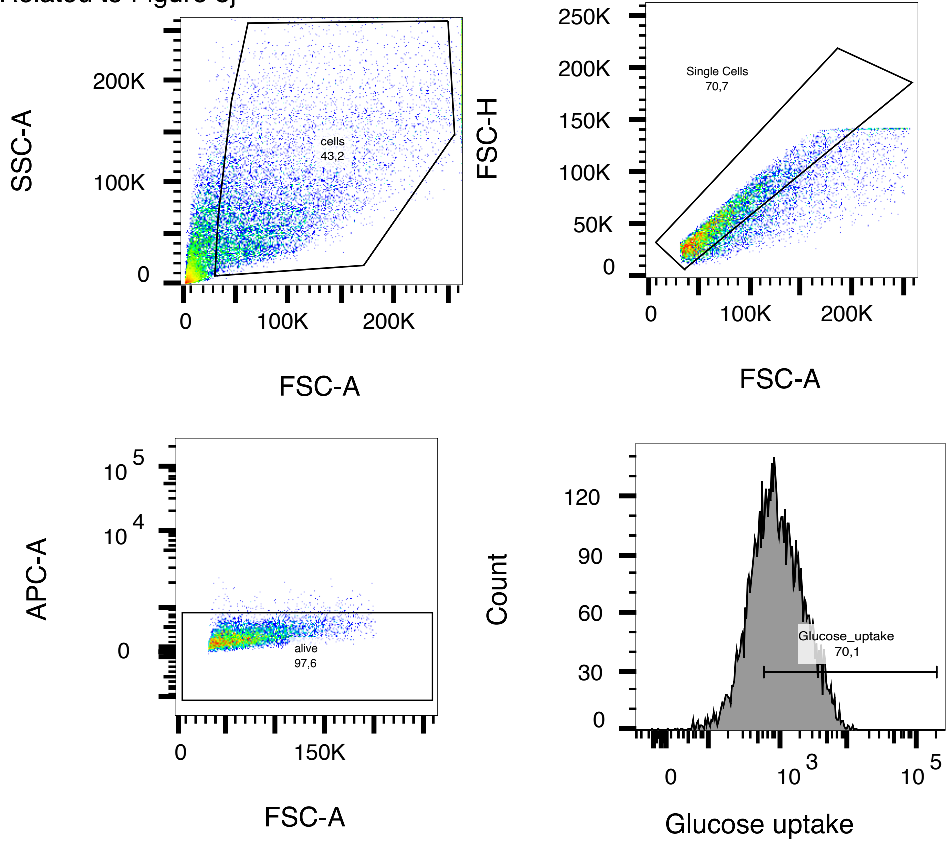

Related to Figure 6e and 7d

iAdipo

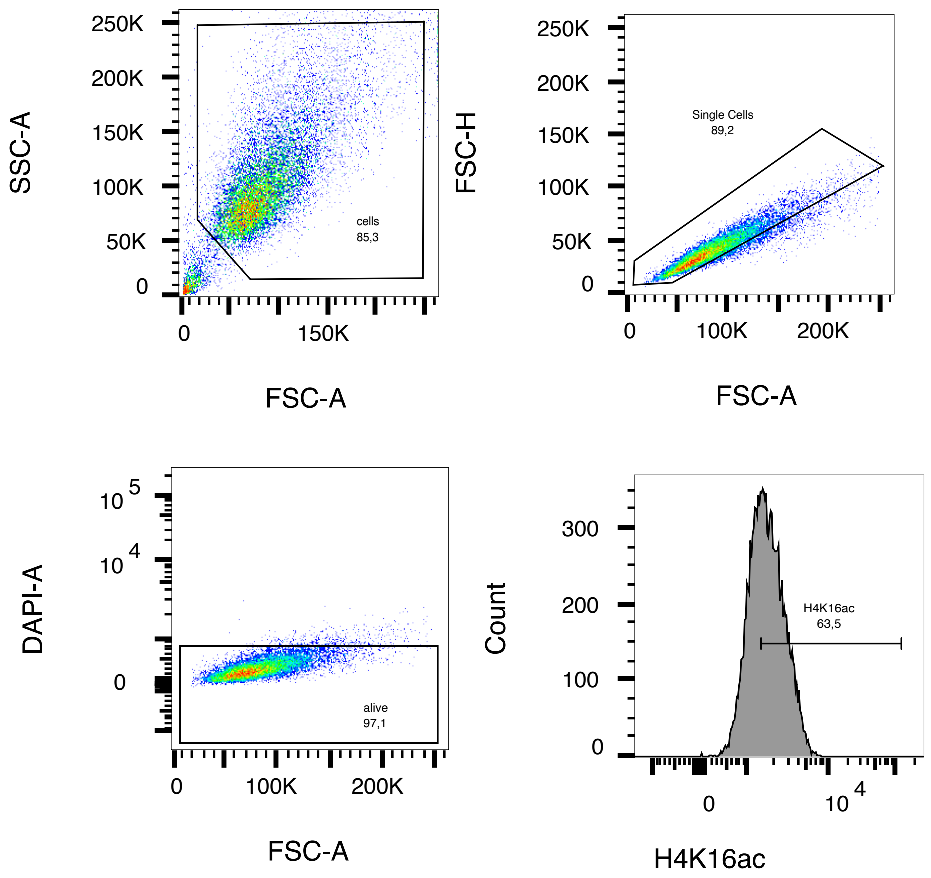

Related to Figure Supplementary 1b

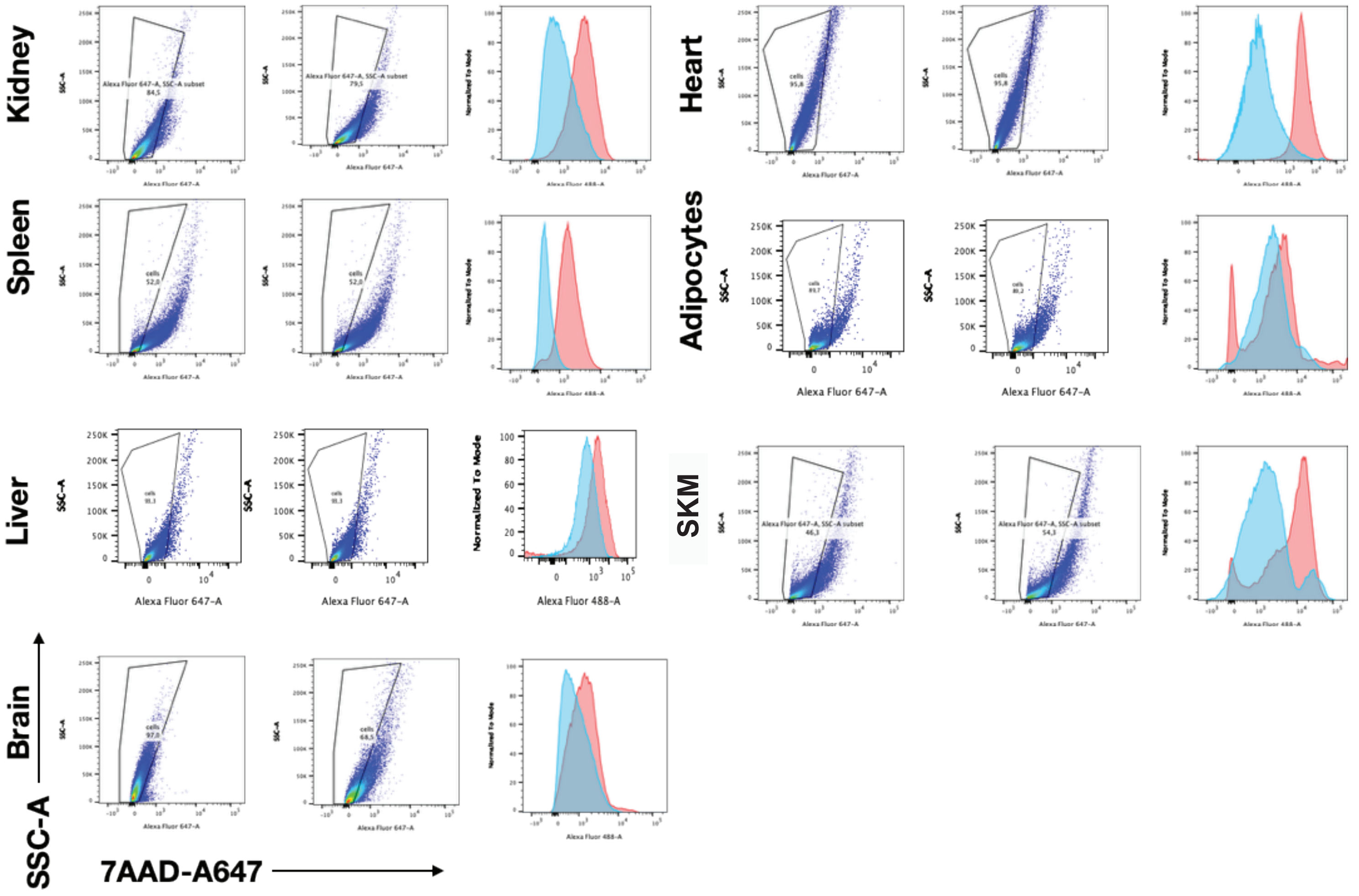

Related to Supplementary Figure 3d

Pancreas

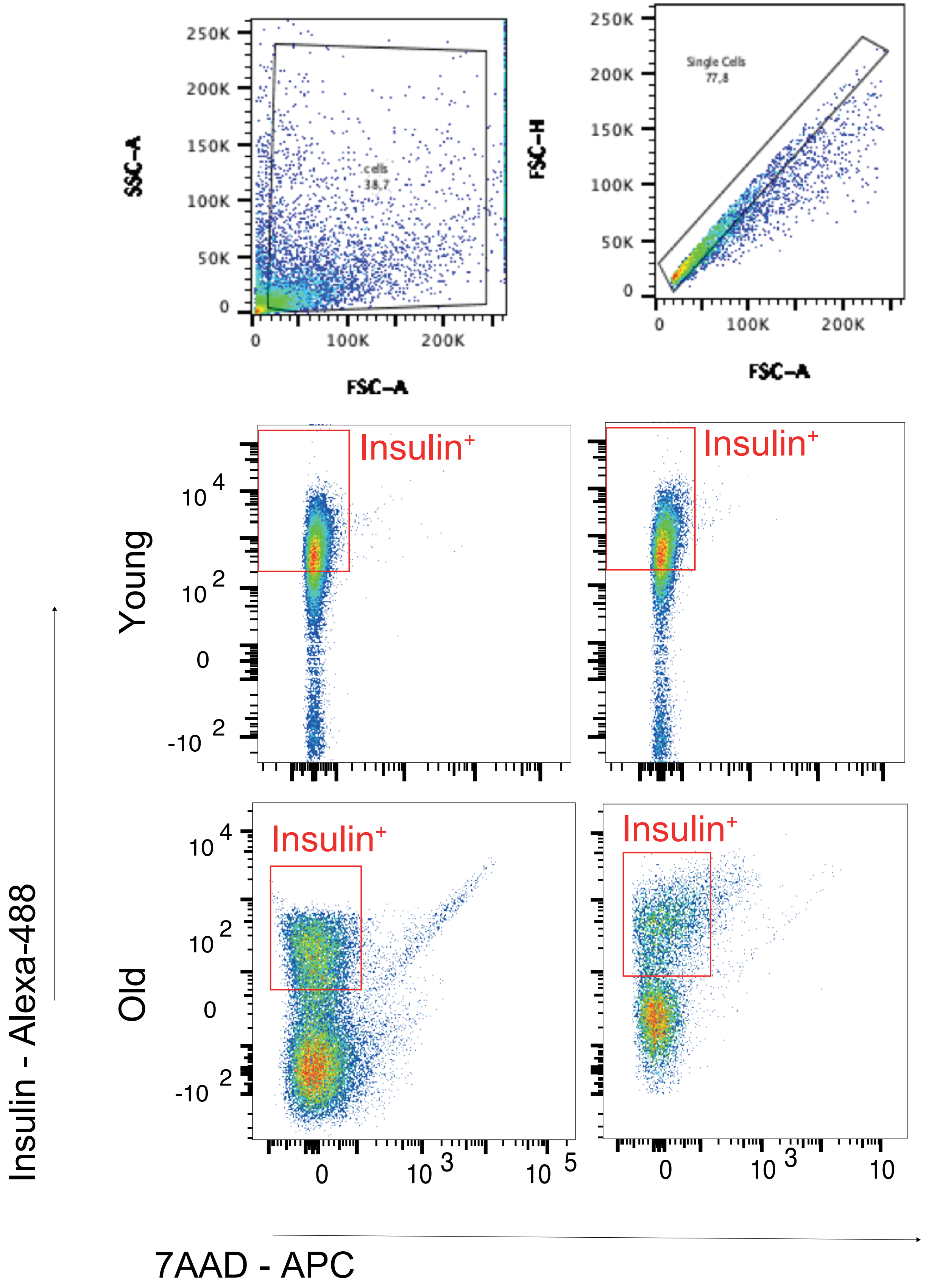

Related to Supplementary Figure 4j

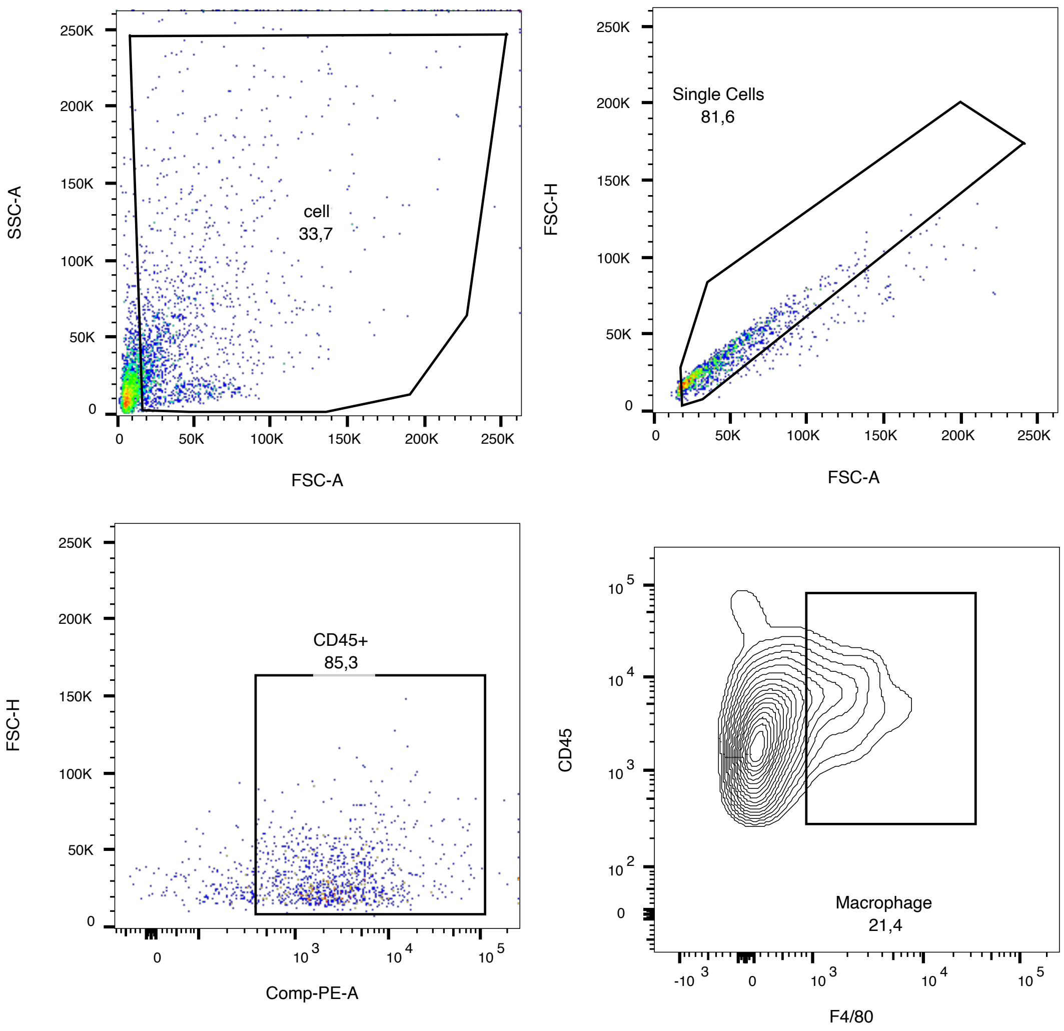

Related to Supplementary Figure 4k and l

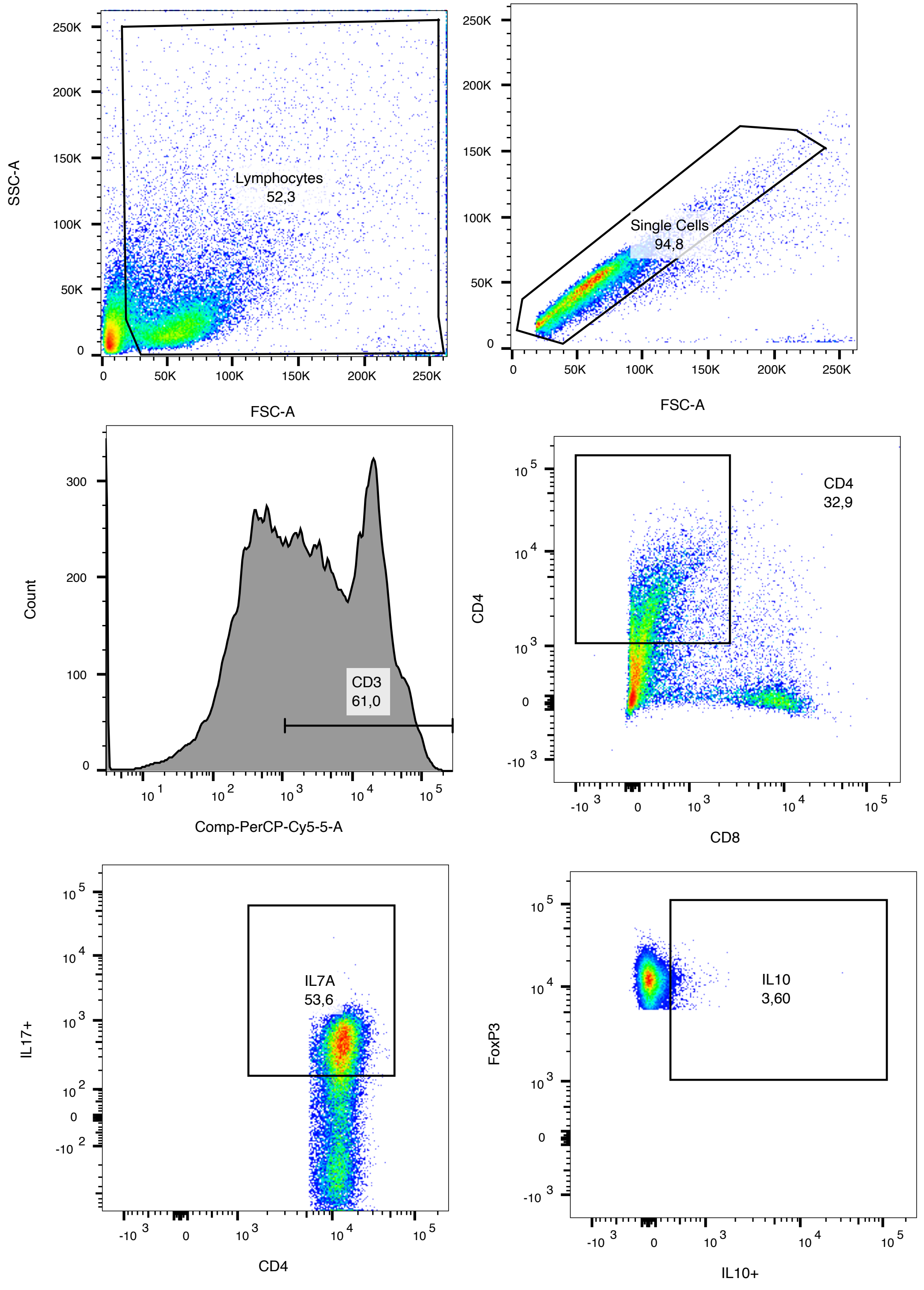

Related to Supplementary Figure 4o

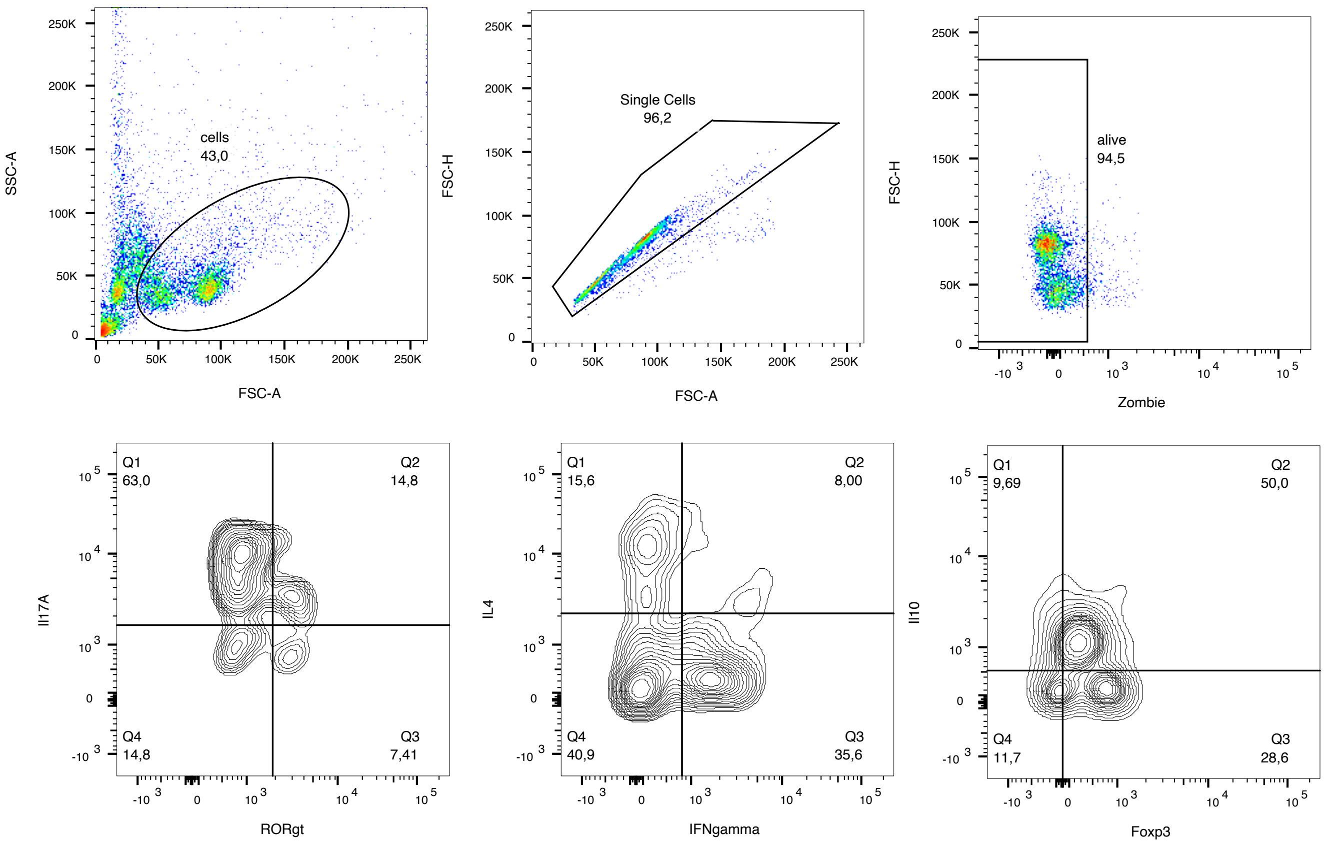

Supplement: Supplementary file 9 — Source Data files [file 41467_2021_26277_MOESM9_ESM.zip › SourceFile_FACS_gating_strategies.pdf]
