# Supplementary material for: Histone H4 lysine 16 acetylation controls central carbon metabolism and diet-induced obesity in mice
Source: Nat Commun. 2021 Oct 27;12:6212. doi: 10.1038/s41467-021-26277-w (PMC8551339; doi:10.1038/s41467-021-26277-w)
Supplement: Supplementary file 9 — Source Data files [file 41467_2021_26277_MOESM9_ESM.zip › SourceFile_western_blots.pdf]

Related to Extended Data Figure 1b

Thymus

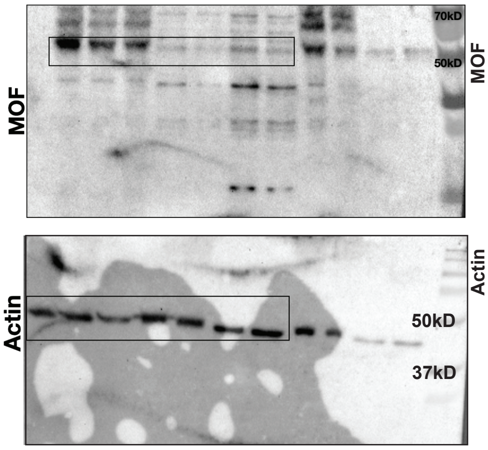

Liver

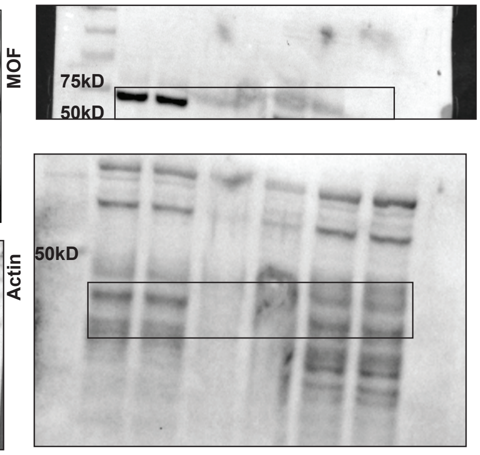

Related to Extended Data Figure 1c

Brain

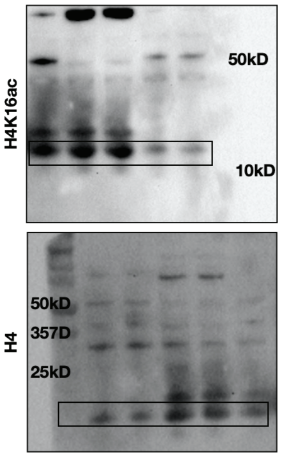

Kidney

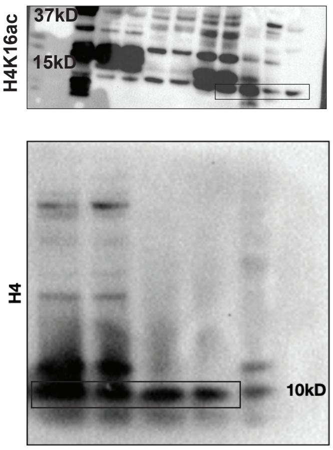

Related to Extended Data Figure 1

Related to Extended Data Figure 6g

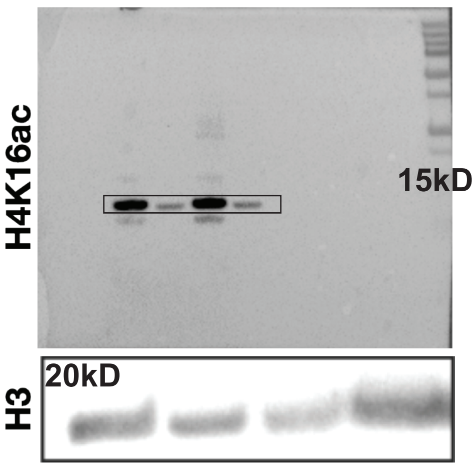

Related to Extended Data Figure 6h

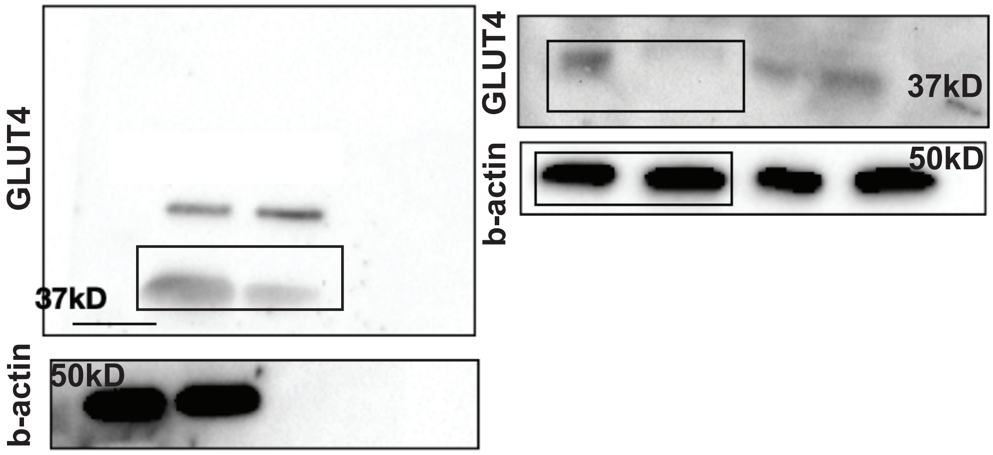

Related to Extended Data Figure 6i

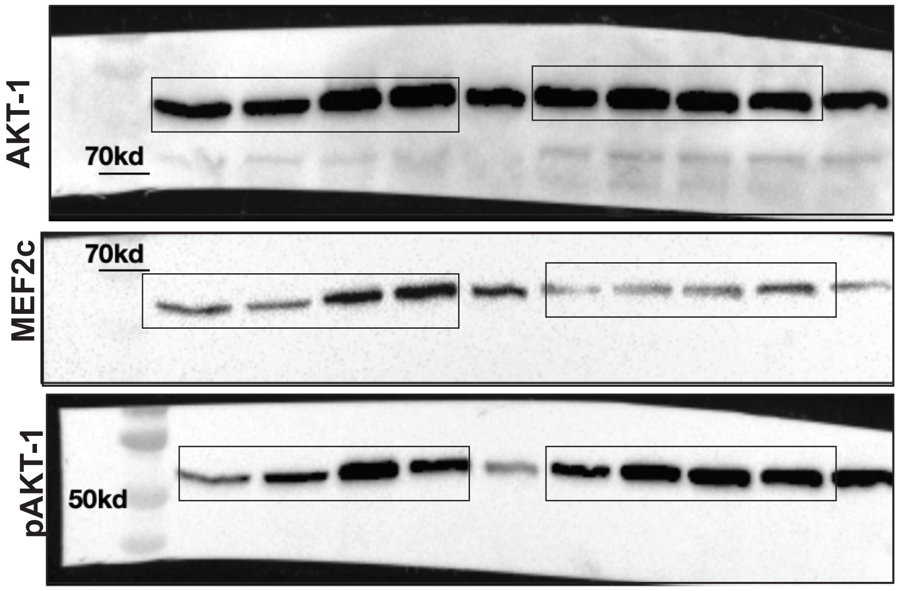

Related to Extended Data Figure 6

Related to Extended Data Figure 7j

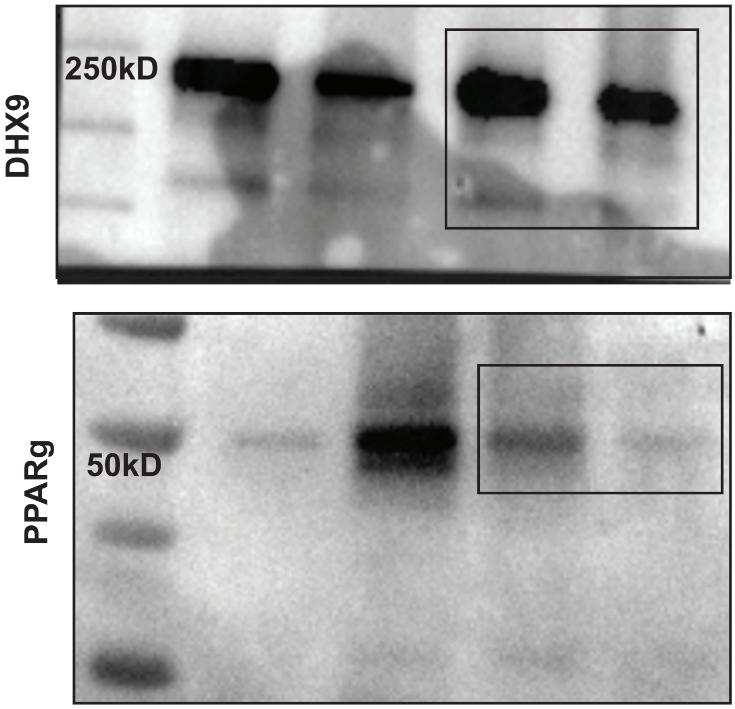

Related to Extended Data Figure 7
